# Mendelian randomization study of micronutrients and development of CKD in a Korean population

**DOI:** 10.1186/s12937-025-01160-2

**Published:** 2025-06-13

**Authors:** Juyeon Lee, Sangjun Lee, Kook-Hwan Oh, Sue K. Park

**Affiliations:** 1 Department of Preventive Medicine, College of Medicine, Seoul National University, 103 Daehakro, Jongnogu, Seoul, Republic of Korea; 2 Cancer Research Institute, Seoul National University, 103 Daehak-Ro, Jongnogu, Seoul, Republic of Korea; 3 Integrated Major in Innovative Medical Science, Seoul National University Graduate School, 103 Daehakro, Jongnogu, Seoul, Republic of Korea; 4 Department of Internal Medicine, Seoul National University Hospital, 103 Daehakro, Jongnogu, Seoul, Republic of Korea; 5 Kidney Research Institute, Seoul National University Medical Research Center, 103 Daehakro, Jongnogu, Seoul, Republic of Korea

**Keywords:** Micronutrients, CKD, GWAS, Mendelian randomization

## Abstract

**Background:**

Although dietary intake is a key modifiable risk factor in the development of chronic kidney disease (CKD), the optimal consumption levels to prevent CKD and the intake levels that pose the least risk remain unclear. Building on the findings from our previous cohort study, this research aims to use genetic variants as instrumental variables to clarify the complex relationship between micronutrient status and the pathogenesis of CKD.

**Methods:**

Of 5,078 participants with a baseline estimate glomerular filtration rate (eGFR) ≥ 60 mL/min/1.73 m^2^ and who were not diagnosed with CKD, we ascertained 708 new CKD cases over 12 year follow-up periods. Mendelian randomization analyses were conducted using genetic instrumental variables to examine the causal relationships between dietary micronutrients (Phosphorus, Vitamin B2, B6 and C) levels and the development of CKD.

**Results:**

In Mendelian randomization study, using the inverse variance-weighted (IVW) radial method, dietary vitamin B6 (β = -4.016, *p*-value = 8.72E-05) and C (β = 2.573, *p* = 1.41E-05) intake levels demonstrated significant associations with the development of CKD. However, there was no significant association observed for dietary phosphorus and vitamin B2 intake levels with the development of CKD (*p* > 0.05).

**Conclusions:**

This study found a weak causal link to genetically predicted levels of vitamins B6 and C on CKD development. Given potential residual pleiotropy and biological limitations, findings should be cautiously interpreted yet highlight the possible role of balanced micronutrient intake in kidney health.

**Supplementary Information:**

The online version contains supplementary material available at 10.1186/s12937-025-01160-2.

## Background

Chronic Kidney Disease (CKD) represents a significant global health challenge, with its increasing prevalence and associated adverse outcomes, including elevated morbidity and mortality rates [1, 2]. The complexity of CKD pathogenesis involves a myriad of factors, among which micronutrients have received substantial attention. Micronutrients, such as vitamins and minerals, play crucial roles in various physiological processes, including immune function, oxidative stress modulation, and metabolic regulation, which are vital for maintaining overall health and potentially influencing CKD development and management [3, 4].

Phosphorus is essential for bone health and cellular function, yet its excess has been implicated in CKD development. By examining genetic proxies for phosphorus intake, we aim to clarify its role in CKD development [5]. Riboflavin (vitamin B2) is crucial for energy production and cellular function, and investigating its causal relationship with CKD could provide insights into metabolic factors contributing to kidney health [6]. Vitamin B6 is involved in protein metabolism and immune function, and understanding its causal link with CKD may reveal important aspects of nutrient metabolism affecting kidney disease [7]. Vitamin C, a potent antioxidant, plays a role in reducing oxidative stress and inflammation, and exploring its causal effect on CKD could highlight its potential as a preventive or therapeutic agent [8].

Observational studies have suggested potential links between micronutrient levels and CKD outcomes [9, 10]. However, these studies often encounter difficulties in establishing causal relationships due to the presence of confounding variables and the inherent issue of reverse causation. Confounding factors, such as dietary habits, lifestyle choices, and pre-existing health conditions, can influence both micronutrient levels and CKD risk, making it challenging to discern the true nature of these associations. To address these limitations, Mendelian randomization (MR) analysis has emerged as a robust methodological approach [11]. MR uses genetic variants as instrumental variables to determine causal relationships between exposures (e.g., micronutrient levels) and outcomes (e.g., CKD risk). As genetic variants are randomly assigned at conception and are not influenced by confounding factors, MR offers a more reliable method for exploring causality compared to traditional observational studies [12]. The selection of phosphorus, vitamin B2, vitamin B6, and vitamin C as exposures in this study was guided by evidence from our previous prospective cohort study, which reported significant associations between dietary intake of these micronutrients and the risk of developing CKD [9]. These findings provided the rationale for including these four micronutrients in the present Mendelian randomization analysis to explore their potential causal roles in CKD pathogenesis.

This study aims to utilize MR analysis to investigate the potential causal effects of several key micronutrients—phosphorus, vitamin B2, vitamin B6, and vitamin C— on the risk of developing CKD. By employing genetic variants associated with these micronutrients as instrumental variables, we seek to disentangle the complex interactions between micronutrient status and CKD development.

## Methods

### Data source and study population

**Fig. 1:**
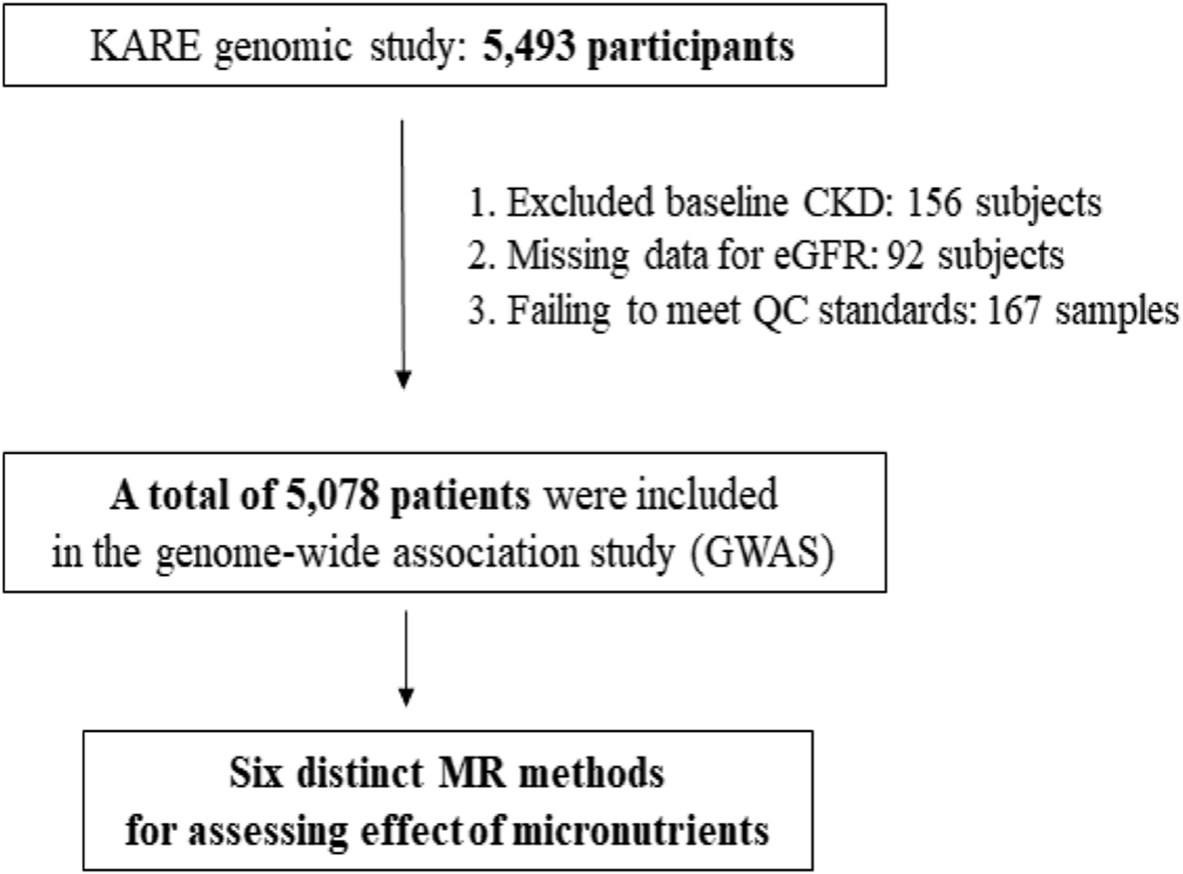
Flow chart of the study population selection from the KARE GWAS and MR study. KARE, Korean Association Resource; GWAS, genome-wide association study; MR, Mendelian randomization

Information regarding KoGES can be found in the previous cohort profile article published by the KCDC (Korea Centers for Disease Control and Prevention) [13]. After obtaining ethical clearance, we acquired survey data and genomic information from the KCDC for our analysis. For our analysis, we utilized cohort data from the KARE project. The KARE project, which began in 2007, is a substantial cohort study that recruited two population samples from the rural Anseong and urban Ansan cohorts [14]. Among the 5,493 participants in the KARE cohort study, 415 subjects were excluded due to missing data on exposure variables (genotyping, nutritional data) and failure to meet quality control standards. The final analysis included 5,078 subjects (Fig. 1). To investigate the association between dietary micronutrient intake levels and the development of CKD in a Korean population, we selected GWAS summary statistics for dietary micronutrient intake levels through a meta-analysis from the Korean Biobank Array (KoGES PheWeb, available at https://koges.leelabsg.org/, assessed January 5, 2024). This analysis included 584,061 SNPs from 708 new CKD cases and 4,370 controls. The K-CHIP consortium, a project initiated by the Center for Genome Science at the National Institute of Health in Korea, includes a specially curated panel of around 830,000 SNPs tailored for the Korean population (4845–301, 3000–3031) [15]. This custom SNP array was developed to enhance genetic research and its relevance to the Korean demographic. Comprehensive procedures for quality control and the methodologies for imputation within the K-CHIP consortium have been thoroughly documented in previous studies [16].

All procedures performed in studies involving human participants were in accordance with the ethical standards of the institutional and/or national research committee and with the 1964 Helsinki declaration and its later amendments or comparable ethical standards. The National Institute of Health, Korea (IRB number 4845–301, 3000–3031) and the Institutional Review Boards of Seoul National University Hospital (C-1704–025–842 and 2101–087–1188) approved this study.

### Outcome variables

CKD was defined as having an estimated glomerular filtration rate (eGFR) below 60 mL/min/1.73 m^2^, aligning with the clinical practice guideline of the National Kidney Foundation Kidney Disease Outcomes Quality Initiative (NKF KDOQI) [17]. A new case of CKD was identified as an eGFR decline to less than 60 mL/min/1.73 m^2^ during a follow-up period spanning from 2 to up to 12 years among cohort members initially presenting with an eGFR exceeding 60 mL/min/1.73 m^2^ at baseline. The eGFR was computed using the CKD-EPI equation, represented as Equation [18].

$$\text{eGFR}=141\times \text{min}{(\frac{Scr}{k})}^{\alpha }\times \text{max}{(\frac{Scr}{k})}^{-1.209}\times {0.993}^{Age}\times 1.018\left[if female\right]$$

Scr = serum creatinine (mg/dL);

k = 0.7 if female; 0.9 if male;

α =  − 0.329 if female; − 0.411 if male;

min = the minimum of Scr/k or 1;

max = the maximum of Scr/k or 1.

### Exposure variables

#### Measurements

Daily nutrient intakes were evaluated utilizing a validated, semi-quantitative Food Frequency Questionnaire (FFQ) specifically designed for the KoGES [19]. Participants were requested to estimate both the average serving size of 106 food items and their consumption frequency. Dietary intakes per day were determined by amalgamating the frequency of servings and portion sizes for each food item with the mean amount per serving. A previous study evaluated the validity and reliability of the FFQ used in the KoGES dataset employed in this research [19]. To assess the reproducibility and validity of the FFQ, it was administered twice at a one-year interval, and three-day dietary records (DR) were collected at each time point for comparison. The analysis revealed correlation coefficients ranging from 0.38 to 0.64 for major nutrient intakes, suggesting that the FFQ is a reliable tool for assessing dietary intake among Korean adults.

All specimens collected were promptly transferred to the Korean National Biobank, ensuring the previously documented reliability of biomarker analyses [16]. Blood and urine samples were acquired using a serum separator tube (SST) and a two-ethylene diamine tetra acetic acid (EDTA) tube, along with a 10 mL midstream urine sample. Post-collection, both serum and plasma underwent meticulous preparation and aliquoting. Blood DNA extraction yielded amounts ranging from 100 to 800 µg, with 6–10 vials (300–500 µL per vial) carefully prepared. Each tube was tagged with a two-dimensional barcode for precise identification. Serum creatinine levels were assessed using the Jaffe method, employing a HITACHI Automatic Analyzer 7600 (Hitachi, Tokyo, Japan), and an ADVIA 1650 Auto Analyzer (Siemens, Washington, DC, USA). The methodologies utilized in sample processing and analysis to established standards, ensuring the robustness and accuracy of the collected data in accordance with rigorous medical research practices.

### Genotyping and quality control

Genomic DNA was isolated from venous blood samples and genotyped using the Affymetrix Axiom™ KORV1.0–96 Array (Affymetrix, Santa Clara, CA, USA), with 200 ng of genomic DNA utilized for the process. Quality control was conducted using the PLINK program (version 1.9; Free Software Foundation Inc., Boston, MA, USA), leading to the exclusion of samples exhibiting sex inconsistencies or having a call rate below 97%. SNPs were filtered based on criteria such as a call rate below 95%, a minor allele frequency lower than 1%, and significant deviation from the Hardy–Weinberg equilibrium permutation test (*P* < 5 × 10^−5^).

### Assumption of one sample mendelian randomization

One-sample MR analyses were performed to investigate the existence of a causal relationship between micronutrients (dietary phosphorus, vitamin B2, B6 and C) and development of CKD. MR necessitates that genetic instruments are associated with a modifiable exposure of interest (assumption 1), and any relationship between the instruments and outcome is mediated through the exposure (assumption 2). The instrumental variable must affect the outcome only via its effect on the exposure (assumption 3).

The MR results were obtained using six methods: Inverse Variance Weighted (IVW), MR-Egger, Weighted Median, Penalized Weighted Median, Simple Median, and Radial IVW.

1. IVW: The IVW method combines effect estimates from genetic instruments by assigning weights proportional to the inverse of their variances. This approach is highly efficient and statistically robust when horizontal pleiotropy is absent. However, it assumes no intercept in the model, which may limit its reliability if pleiotropic effects are present.
2. MR-Egger: The MR-Egger method incorporates an intercept term into the model to address potential directional pleiotropy, allowing for variations in pleiotropic effects across genetic instruments. While this method is particularly effective in managing heterogeneity, it tends to have lower statistical power compared to the IVW method.
3. Weighted Median: The Weighted Median method generates consistent causal effect estimates, even when up to 50% of the genetic instruments are invalid, provided the instrument strength independent of direct effects (InSIDE) assumption is met.
4. Penalized Weighted Median: This technique extends the Weighted Median approach by introducing penalties for outlier SNPs with significant deviations. By minimizing the impact of such outliers, it enhances the robustness of causal effect estimates and ensures greater reliability.
5. Simple Median: The Simple Median method calculates the causal effect based on the median value of individual SNP effect estimates. Although it is less precise than the Weighted Median or Penalized Weighted Median, it provides a viable option when the number of valid instruments is small.
6. Radial IVW: Radial IVW modifies the standard IVW approach by using a radial regression framework to identify and adjust for outlier SNPs that contribute disproportionately to heterogeneity. This method is particularly useful for addressing complex pleiotropic effects, resulting in more refined and reliable effect estimates [20].

### Statistical analysis

**Fig. 2:**
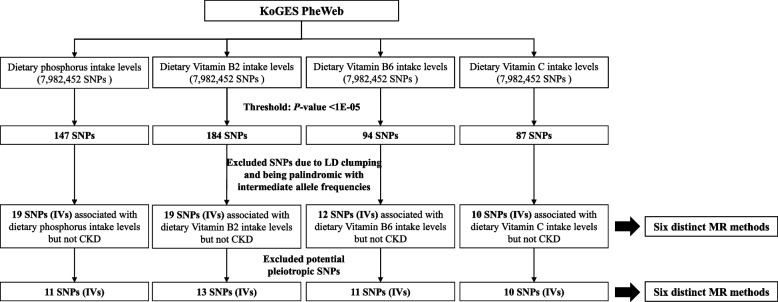
Overview of the selection IVs associated with dietary micronutrient levels but not CKD for MR analysis. KoGES, the Korean genome and epidemiology study; SNPs, single nucleotide polymorphism; LD, linkage disequilibrium; CKD, chronic kidney disease; IV, instrumental variable; MR, Mendelian randomization

From the GWAS summary statistics encompassing 7,982,452 SNPs related to dietary micronutrient levels from the KoGES PheWeb, SNPs meeting a suggestive significance threshold of *p* < 1 × 10⁻^5^ were identified as statistically significant (Fig. 2). Given that GWAS-summary statistics were analyzed independently for each dietary micronutrient (phosphorus, vitamin B2, vitamin B6, and vitamin C), multiple testing correction across these micronutrients was not applied. Each nutrient was considered as an independent exposure, thus mitigating the risk associated with multiple comparisons across different exposures. Specifically, 147 SNPs for phosphorus, 184 SNPs for vitamin B2, 94 SNPs for vitamin B6, and 87 SNPs for vitamin C were selected based on statistical significance. SNPs were subsequently filtered through LD clumping and exclusion of palindromic SNPs with intermediate allele frequencies (Fig. 2). SNPs unrelated to CKD were further selected as instrumental variables (IVs) for each dietary micronutrient, resulting in 19 IVs for phosphorus, 19 for vitamin B2, 12 for vitamin B6, and 10 for vitamin C (Fig. 2). For additional sensitivity analysis, previously reported mapped phenotypes for each SNP were examined through the GWAS Catalog (https://www.ebi.ac.uk/gwas/), aiming to identify potential pleiotropic SNPs. After this process, final IV selections included 11 SNPs for phosphorus, 13 for vitamin B2, 11 for vitamin B6, and 10 for vitamin C (Fig. 2).

MR analysis was performed using genetic instrumental variables to evaluate the causal relationship between dietary micronutrient levels and the development of CKD. To ensure a robust assessment of the association between dietary micronutrients and CKD risk, we employed six distinct methods (MR Egger regression, Inverse Variance Weighted, Simple median, Weighted Median, Penalized Weighted Median, and Inverse Variance Weighted Radial method) during the MR analysis [20].

To validate the associations between the selected IVs and each dietary micronutrient level, polygenic risk scores (PRS) were constructed based on the selected IVs and their corresponding effect sizes. Linear regression models were performed to evaluate associations between the PRS and actual dietary micronutrient intake levels. The PRS was estimated using the following formula [21]:

$$PRS= {\sum }_{i=1}^{n}{\beta }_{i}\times {SNP}_{i} ({SNP}_{i} consided as {IV}_{i})$$

where ${\beta }_{i}$ is the effect size for the ${i}^{th}$ SNP obtained from GWAS and ${SNP}_{i}$ is the genotype dosage of the ${i}^{th}$ SNP.

Additionally, in our study, we employed the Mendelian Randomization Pleiotropy RESidual Sum and Outlier (MR-PRESSO) method as part of the Mendelian Randomization analysis to assess the causal relationships between micronutrient levels and CKD development [22]. MR-PRESSO is a robust statistical approach designed to detect and correct for horizontal pleiotropy, which occurs when genetic variants used as instrumental variables affect the outcome through pathways other than the exposure of interest. This method enhances the reliability of causal inferences by addressing potential biases introduced by pleiotropic effects.

## Results

### General characteristics

The CKD development group exhibited characteristics such as older age, a higher proportion of females, and increased prevalence of diabetes and hypertension. Non-smokers were more common in the non-CKD group, with no significant difference in physical activity levels observed between the groups. Anthropometrically, individuals with CKD development had a higher mean BMI, elevated blood pressure readings, and elevated serum creatinine levels. Additionally, HDL cholesterol levels were lower in the CKD development group. In terms of dietary habits, the CKD development group generally demonstrated lower intake of minerals and vitamins, with the exception of vitamin C, where intake was higher in the CKD development group Table 1.

**Table 1:** General characteristics of the study subjects

**New CKD cases at follow-up from 2 to 12 years**			***P*-value**^b^
	**CKD development**^a^	**Non-CKD**^a^	
	*N* = 708	*N* = 4,370	
	**Mean (SD)**	**Mean (SD)**	
**Dietary micronutrients levels**			
Dietary minerals			
Calcium intake (mg/day)	459.7 (267.4)	485.5 (270.1)	0.02
Phosphorus intake (mg/day)	998.0 (410.4)	1039.3 (419.3)	0.01
Sodium intake (mg/day)	3232.1 (1688.8)	3210.5 (1637.8)	0.75
Potassium intake (mg/day)	2528.9 (1219.2)	2566.0 (1198.6)	0.44
Iron intake (mg/day)	10.7 (5.0)	11.1 (5.4)	0.04
Zinc intake (mg/day)	8.6 (5.4)	9.0 (4.5)	0.08
Dietary vitamins			
Vitamin A intake (μg/day)	505.2 (345.8)	546.5 (411.9)	0.01
Retinol intake (μg/day)	60.4 (69.4)	70.0 (61.3)	< 0.01
Carotene intake (μg/day)	2606.7 (1964.5)	2796.9 (2403.0)	0.04
Vitamin B1 intake (mg/day)	1.2 (0.5)	1.3 (0.6)	0.01
Vitamin B2 intake (mg/day)	0.9 (0.5)	1.0 (0.5)	< 0.01
Niacin intake (mg/day)	14.8 (6.3)	16.0 (7.0)	< 0.01
Vitamin B6 intake (mg/day)	1.8 (0.8)	1.8 (0.8)	0.04
Folate intake (μg/day)	246.1 (129.1)	249.9 (132.4)	0.47
Vitamin C intake (mg/day)	133.1 (97.9)	129.2 (97.8)	0.32
Age (years)	59.3 (7.7)	50.6 (8.3)	< 0.01
Body mass index (kg/mg^2^)	25.1 (3.4)	24.5 (3.1)	< 0.01
Systolic blood pressure (mmHg)	128.6 (20.6)	119.0 (18.1)	< 0.01
Diastolic blood pressure (mmHg)	82.8 (11.7)	79.2 (12.1)	< 0.01
Serum creatinine (mg/dL)	0.9 (0.2)	0.8 (0.2)	< 0.01
Serum hemoglobin (g/dL)	13.5 (1.4)	13.6 (1.6)	0.07
HDL cholesterol (mg/dL)	48.5 (11.9)	49.9 (11.9)	< 0.01
	***N* (%)**	***N* (%)**	
Sex			< 0.01
Male	277 (39.1)	2128 (48.7)	
Female	431 (60.9)	2242 (51.3)	
Smoking status			0.01
No	452 (63.8)	2531 (57.9)	
Yes	249 (35.2)	1805 (41.3)	
Physical activity			0.30
No	362 (51.1)	2111 (48.3)	
Yes	336 (47.5)	2177 (49.8)	
Diabetes mellitus^c^			< 0.01
No	574 (81.1)	4058 (92.9)	
Yes	134 (18.9)	312 (7.1)	
Hypertension^d^			< 0.01
No	425 (60.0)	3240 (74.1)	
Yes	283 (39.9)	1130 (25.9)	

^a^CKD was defined as a ‘eGFR < 60 mL/min/1.73 m2’, in compliance with the National Kidney Foundation Kidney Disease Outcomes Quality Initiative (NKF KDOQI) clinical practice guideline. A new case of CKD was defined as a eGFR decline to < 60 mL/min/1.73 m2 over a follow-up period ranging from 2 to up to 12 years among cohort members with an eGFR greater than 60 mL/min/1.73 m2 at the time of baseline

^b^For continuous variable, T-test was used. For categorical variables, chi-square test was used

^c^DM defined as history of diabetes mellitus or elevated plasma glucose ≥ 100 mg/dl or taking anti-diabetic medications

KARE, Korea Association resource

^d^HTN defined as history of hypertension, SBP > 130 mmHg or DBP > 85 mmHg or taking anti-hypertensive medications

### GWAS study

After undergoing data quality control processes, we present in Supplementary Tables 1 to 4 the SNPs associated with dietary micronutrients, including phosphorus, vitamin B2, B6, and C. These tables include information such as chromosome number, position within the chromosome, SNP name, nearest gene, alleles, minor allele frequency (MAF), beta coefficient, standard error (SE), and *p*-value. A total of 62 SNPs were identified for dietary phosphorus intake, 56 SNPs for vitamin B2, 66 SNPs for vitamin B6, and 50 SNPs for vitamin C, all of which exhibited a *p*-value < 1e-5. Furthermore, we validated the results of the GWAS analysis through the utilization of a Manhattan plot (Supplementary Fig. 1 to 4).

### MR analysis

The summary statistics for SNPs selected as IVs for each dietary micronutrient, including beta coefficients, SEs, *p*-values, risk alleles, and risk allele frequencies, are presented in Supplementary Tables 5–8. After excluding SNPs identified as potential sources of pleiotropy, summary statistics for the remaining SNPs utilized as IVs are detailed in Tables 2, 3, 4 and 5.

**Table 2:** Associations of individual genetic instruments for dietary phosphorus intake levels with CKD development, after excluding potential pleiotropic SNPs

CHR	SNP	Nearest Genes	Mapped phenotypes	Effect allele	MAF	Dietary phosphorus intake levels			CKD development		
						**Beta**	**SE**	***P*-value**	**Beta**	**SE**	***P*-value**
17	rs8074317	SEPTIN9	Phosphorus, Vitamin B2 intake	T	0.220	−0.021	0.0036	6.00E-09	−0.024	0.099	0.806
14	rs138424249	LRFN5	Phosphorus intake	A	0.092	0.029	0.0054	6.20E-08	−0.179	0.145	0.218
7	rs930110	FZD1	Phosphorus intake	G	0.530	0.015	0.003	2.50E-07	0.019	0.103	0.850
2	rs10193255	RBM43	Phosphorus intake	C	0.330	−0.016	0.0032	4.20E-07	0.032	0.084	0.700
8	rs77133047	DOK2	Phosphorus intake	T	0.180	−0.019	0.0039	1.10E-06	−0.153	0.129	0.234
11	rs7126868	PRMT3	Phosphorus intake	C	0.340	0.015	0.0032	1.50E-06	0.014	0.101	0.889
18	rs78281436	MYO5B	Phosphorus intake, HDL	C	0.046	−0.036	0.0075	1.50E-06	0.202	0.215	0.347
5	rs145817449	MROH2B	Phosphorus intake, Gastric cancer	C	0.018	−0.053	0.011	2.70E-06	−0.307	0.280	0.272
11	rs365215	PLEKHA7	Phosphorus intake, BMI	C	0.280	0.016	0.0034	3.30E-06	0.090	0.104	0.385
4	rs2567388	EMCN	Phosphorus intake, Hyperlipidemia	T	0.300	−0.015	0.0033	4.70E-06	−0.067	0.084	0.427
10	rs7902081	HMX3	Phosphorus intake	C	0.130	−0.022	0.0049	5.40E-06	−0.070	0.131	0.595

*SNP* Single nucleotide polymorphism, *MAF* Minor allele frequency, *SE* Standard error, *WC* Waist circumference, *TC* Total cholesterol, *HDL* High-Density Lipoprotein, *BMI* Body mass index, *DM* Diabetes mellitus, *LDL* Low-Density Lipoprotein

**Table 3:** Associations of individual genetic instruments for dietary vitamin B2 intake levels with CKD development, after excluding potential pleiotropic SNPs

CHR	SNP	Nearest Genes	Mapped phenotypes	Effect allele	MAF	Dietary vitamin B2 intake levels			CKD development		
						**Beta**	**SE**	***P*-value**	**Beta**	**SE**	***P*-value**
17	rs8074201	SEPTIN9	Vitamin B2, Phosphorus intake	T	0.220	−0.024	0.004	3.90E-08	−0.024	0.099	0.804
1	rs147957210	PRKCZ	Vitamin B2 intake, BMI	G	0.032	−0.048	0.010	1.60E-06	−0.349	0.239	0.144
13	rs141095648	NUFIP1	Vitamin B2, Sodium intake	A	0.075	−0.032	0.007	2.80E-06	0.004	0.156	0.980
3	rs17018468	LRRC3B	Vitamin B2, Iron, Calcium intake	G	0.290	−0.018	0.004	3.20E-06	−0.037	0.087	0.667
5	rs1974852	EMB	Vitamin B2 intake, DM	A	0.170	−0.023	0.005	3.40E-06	0.008	0.110	0.940
3	rs80277692	NMD3	Vitamin B2 intake, Gastric cancer	G	0.140	−0.024	0.005	3.60E-06	−0.104	0.117	0.373
2	rs1485984	KCNH7	Vitamin B2 intake, Creatinine	T	0.380	0.017	0.004	4.20E-06	0.074	0.082	0.368
10	rs72822548	SORBS1	Vitamin B2 intake, RBC	A	0.370	−0.017	0.004	4.30E-06	−0.116	0.085	0.174
2	rs10193255	RBM43	Vitamin B2, Phosphorus intake	C	0.330	−0.017	0.004	6.40E-06	0.032	0.084	0.700
7	rs1346667	FZD1	Vitamin B2, Phosphorus, Calcium intake	G	0.200	0.020	0.005	7.20E-06	0.029	0.128	0.820
9	rs10973705	SHB	Vitamin B2, Calcium intake	T	0.470	−0.016	0.004	8.70E-06	−0.133	0.081	0.100
4	rs185500746	FBXW7	Vitamin B2 intake, DM	C	0.024	−0.051	0.011	8.70E-06	−0.080	0.253	0.750
6	rs146438270	LRFN2	Vitamin B2 intake, BMI	T	0.020	−0.061	0.014	9.60E-06	0.038	0.358	0.915

*SNP* Single nucleotide polymorphism, *MAF* Minor allele frequency, *SE* Standard error, *RBC* Red blood cell, BMI Body mass index, *DM* Diabetes mellitus

**Table 4:** Associations of individual genetic instruments for dietary vitamin B6 intake levels with CKD development, after excluding potential pleiotropic SNPs

CHR	SNP	Nearest Genes	Mapped phenotypes	Effect allele	MAF	Dietary vitamin B6 intake levels			CKD development		
						**Beta**	**SE**	***P*-value**	**Beta**	**SE**	***P*-value**
3	rs145928548	NSUN3	Vitamin B6 intake	T	0.015	−0.072	0.014	9.20E-08	0.319	0.351	0.363
5	rs184559817	HMHB1	Vitamin B6 intake, Height	A	0.010	0.088	0.017	1.50E-07	−0.421	0.472	0.372
3	rs148237512	NSUN3	Vitamin B6 intake	T	0.015	−0.069	0.014	5.30E-07	0.450	0.369	0.223
19	rs77055181	NPHS1	Vitamin B6, Iron intake, Creatinine	A	0.086	−0.029	0.006	7.10E-07	0.027	0.160	0.867
9	rs4742795	CAVIN4	Vitamin B6, Niacin intake	A	0.380	0.017	0.004	8.90E-07	−0.091	0.084	0.279
10	rs141302176	ADAM12	Vitamin B6, Niacin, Iron intake	A	0.026	−0.048	0.010	2.10E-06	0.169	0.243	0.486
1	rs2071987	VAMP3	Vitamin B6, Iron, Folate intake	A	0.390	0.015	0.003	5.60E-06	−0.043	0.087	0.623
1	rs375382680	ATP2B4	Vitamin B6, Fiber, Iron intake	T	0.020	−0.052	0.012	7.40E-06	−0.099	0.294	0.737
6	rs12192239	DLL1	Vitamin B6, Iron, Folate intake	T	0.630	−0.016	0.004	7.90E-06	0.195	0.097	0.045
2	rs35280662	AC007040.2	Vitamin B6, Ash, Sodium intake	A	0.035	−0.042	0.009	8.10E-06	0.217	0.229	0.344
3	rs9289799	PFN2	Vitamin B6, Iron, Niacin intake	G	0.200	0.018	0.004	9.50E-06	−0.015	0.099	0.883

*KARE* Korea Association resource, *SNP* Single nucleotide polymorphism, *MAF* Minor allele frequency, *SE* Standard error

**Table 5:** Associations of individual genetic instruments for dietary vitamin C intake levels with CKD development, after excluding potential pleiotropic SNPs

CHR	SNP	Nearest Genes	Mapped phenotypes	Effect allele	MAF	Dietary vitamin C intake levels			CKD development		
						**Beta**	**SE**	***P*-value**	**Beta**	**SE**	***P*-value**
1	rs140394939	PDE4B	Vitamin C, Carotene intake	A	0.015	−0.098	0.020	1.20E-06	−0.117	0.380	0.758
2	rs16846116	LRP1B	Vitamin C, Weight, BMI	A	0.070	0.044	0.009	1.20E-06	0.129	0.159	0.416
8	rs147902155	SNAI2	Vitamin C, Potassium, Vitamin B2 intake	A	0.029	−0.068	0.014	2.00E-06	−0.308	0.306	0.315
1	rs12031723	PTBP2	Vitamin C, Fiber, Potassium intake	C	0.250	−0.026	0.006	2.90E-06	−0.129	0.101	0.201
5	rs76463900	HDAC3	Vitamin C intake, WBC, Height	A	0.140	0.032	0.007	4.60E-06	−0.041	0.120	0.734
5	rs10074128	PRLR	Vitamin C, A, Folate intake, BUN	G	0.390	0.022	0.005	6.90E-06	0.067	0.083	0.415
7	rs2521745	NPVF	Vitamin C, Fiber, Vitamin B6, Carotene intake	A	0.800	0.026	0.006	7.30E-06	0.036	0.131	0.785
2	rs3828277	TNS1	Vitamin C, Potassium intake, HbA1 C	T	0.065	−0.042	0.009	7.50E-06	−0.101	0.153	0.508
8	rs146782291	C8orf37	Vitamin C, Fiber intake, Colorectal cancer	A	0.044	0.052	0.012	8.90E-06	0.203	0.256	0.427
1	rs898833	STUM	Vitamin C, Vitamin B6, Fiber intake	T	0.230	−0.024	0.006	9.40E-06	−0.083	0.100	0.406

*SNP* Single nucleotide polymorphism, *MAF* Minor allele frequency, *SE* Standard error, *BUN* Blood Urea Nitrogen, *BMI* Body mass index, *WBC* White Blood Cell, *HbA1 C* Glycated Hemoglobin

MR analyses were performed to examine the causal effects of dietary phosphorus, vitamin B2, B6, and vitamin C intake levels on CKD development risk, with primary results focusing on analyses after removing potentially pleiotropic SNPs. Table 6 summarizes these primary MR estimates, while Supplementary Table 9 provides MR estimates obtained from the complete set of SNPs (including potential pleiotropic SNPs).

**Table 6:** Mendelian randomization results for the effects of dietary phosphorus and vitamin B2, B6, and C intake on CKD development, after excluding potential pleiotropic SNPs

Micronutrient levels	SNPs	BETA	SE	*p*-value
**Dietary phosphorus intake levels**				
IVW	11	1.110	1.728	5.20E-01
IVW radial	11	1.111	1.397	4.27E-01
MR Egger	11	0.261	5.139	4.75E-01
Penalised weighted median	11	1.225	2.378	6.06E-01
Weighted median	11	1.225	2.310	5.96E-01
Simple median	11	1.293	2.438	5.96E-01
**Dietary Vitamin B2 intake levels**				
IVW	13	2.637	1.376	5.53E-02
IVW radial	13	2.637	0.904	3.54E-03
MR Egger	13	3.094	3.368	1.72E-01
Penalised weighted median	13	1.567	1.887	4.06E-01
Weighted median	13	1.567	1.791	3.82E-01
Simple median	13	1.578	1.903	4.07E-01
**Dietary Vitamin B6 intake levels**				
IVW	11	−4.014	1.624	1.35E-02
IVW radial	11	−4.016	1.023	8.72E-05
MR Egger	11	−4.477	4.134	1.31E-01
Penalised weighted median	11	−4.377	2.193	4.59E-02
Weighted median	11	−4.377	2.159	4.27E-02
Simple median	11	−4.431	2.235	4.75E-02
**Dietary Vitamin C intake levels**				
IVW	10	2.572	1.275	4.37E-02
IVW radial	10	2.573	0.592	1.41E-05
MR Egger	10	2.092	3.439	5.59E-01
Penalised weighted median	10	2.952	1.621	6.86E-02
Weighted median	10	2.952	1.626	6.94E-02
Simple median	10	2.997	1.538	5.14E-02

*CKD* Chronic kidney disease, *SNP* Singel nucleotide polymorphism, *SE* Standard error, *IVW* Inverse variance weighted, *MR* Mendelian randomization

After excluding potentially pleiotropic SNPs, dietary vitamin B6 intake levels consistently showed significant negative associations with CKD development across various MR methods. Specifically, the IVW method yielded a significant negative association (beta = −4.014, SE = 1.624, *p* = 1.35E-02), and this result was further supported by the IVW radial method (beta = −4.016, SE = 1.023, *p* = 8.72E-05). Additional methods including Penalized Weighted Median, Weighted Median, and Simple Median also demonstrated significant negative associations with *p*-values of 4.59E-02, 4.27E-02, and 4.75E-02, respectively.

For dietary vitamin C intake, after removal of potential pleiotropy, the IVW method indicated a significant positive association with CKD development (beta = 2.572, SE = 1.275, *p* = 4.37E-02), more supported by the IVW radial method (beta = 2.573, SE = 0.592, *p* = 1.41E-05).

**Fig. 3:**
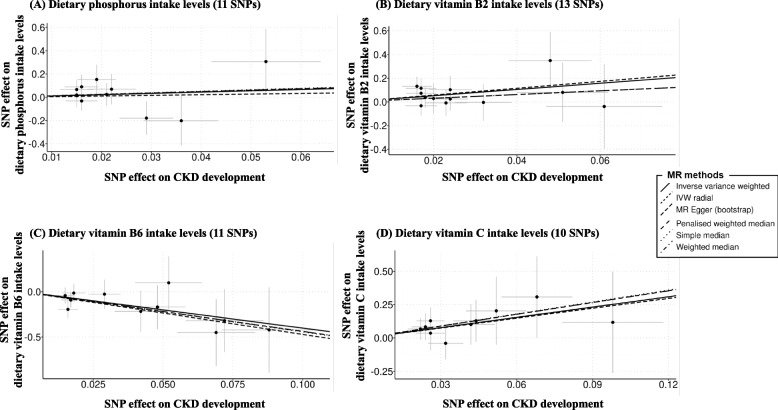
Scatter plots comparing MR analyses of the associations between dietary micronutrient levels and CKD development. Associations between genetic IVs for dietary micronutrient levels (after excluding SNPs with potential pleiotropic effects) and CKD development were analyzed using different MR methods. MR, Mendelian randomization; CKD, chronic kidney disease; IV, instrumental variable; SNP, single nucleotide polymorphism

Figure 3 shows scatter plots of the estimated effects of SNPs (after excluding SNPs with potential pleiotropic effects) on dietary micronutrients levels against the estimated effects of SNPs on the development of CKD. Supplementary Fig. 5 shows scatter plots without excluding potential pleiotropic SNPs, and the results were consistent with those of Fig. 3.

**Fig. 4:**
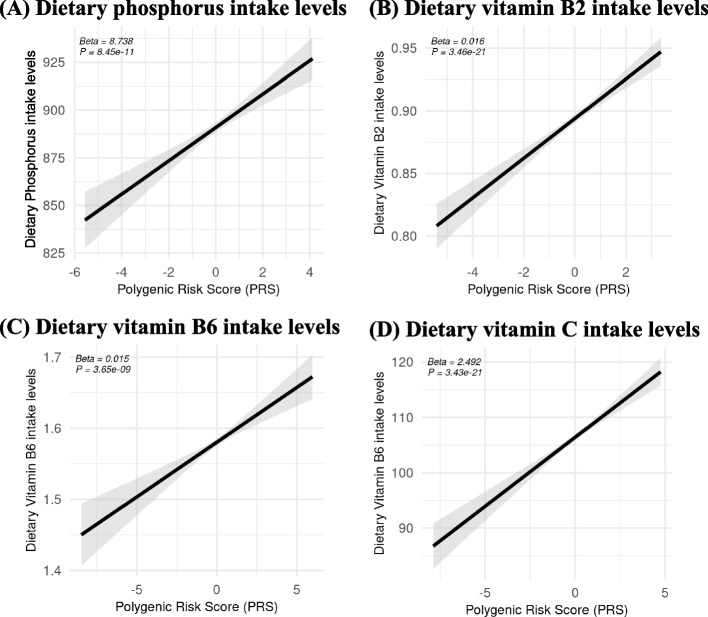
Associations between the PRS derived from IVs—after excluding potential pleiotropic SNPs—and dietary micronutrient levels, based on linear regression models. Black lines indicate beta coefficients, and grey shaded areas represent their 95% CIs. IV, instrumental variable; PRS, polygenic risk score; CI, confidence interval

Supplementary Fig. 6 demonstrates statistically significant associations between PRS derived from IVs and actual dietary micronutrient intake levels based on linear regression models. Specifically, phosphorus (beta = 10.596, *p* = 3.56E-15), vitamin B2 (beta = 0.020, *p* = 1.37E-31), vitamin B6 (beta = 0.016, *p* = 7.30E-10), and vitamin C (beta = 2.492, *p* = 3.43E-21) each showed significant correlations, confirming the validity of the IVs after excluding potential pleiotropic SNPs for these dietary micronutrients (Fig. 4).

Overall, the results suggest a negative causal effect of dietary Vitamin B6 intake on CKD development, while dietary Vitamin C intake levels showed a potential positive association that warrants further investigation. No significant associations were found for dietary phosphorus and Vitamin B2 intake levels.

### Pleiotropy test

The Pleiotropy test was conducted using MR-Egger regression, a sensitivity analysis of the IVW. The intercept in the MR-Egger regression model was estimated to be 0.059 for phosphorus, 0.044 for vitamin B2, −0.026 for vitamin B6, 0.016 for vitamin C with a *p*-value of 0.589, 0.637 0.752 and 0.884, respectively, indicating that there was no directional horizontal pleiotropy (Table 7). Also, the Funnel Plot indicated the absence of direct horizontal pleiotropy in this study (Supplementary Fig. 7). Furthermore, we also conducted the MR-PRESSO global test, yielding *p*-values of 0.779 for phosphorus, 0.779 for vitamin B2, 0.953 for vitamin B6, and 0.895 for vitamin C. These results confirm the absence of vertical pleiotropy (Table 7). Notably, results in Table 7 represent analyses conducted after excluding potential pleiotropic SNPs identified by MR-PRESSO, whereas Supplementary Table 10 presents prior to excluding these potential pleiotropic SNPs.

**Table 7:** Results of pleiotropy test for the association between dietary micronutrients levels and CKD development, after excluding pleiotropic SNPs

**Dietary micronutrients levels**	SNPs, *n*	Intercept	SE	*p*-value	MR-PRESSO *p*-value
Phosphorus	11	0.059	0.106	0.589	0.779
Vitamin B2	13	0.044	0.090	0.637	0.779
Vitamin B6	11	−0.026	0.079	0.752	0.953
Vitamin C	10	0.016	0.111	0.884	0.895

*SNP* Single nucleotide polymorphism

## Discussions

The present study utilized Mendelian randomization analysis to explore the relationship between dietary phosphorus and vitamin B2, vitamin B6 and vitamin C and the risk of CKD development. Based on the findings of this study, there was a weak causal link between genetically predicted levels of vitamins B6 and C and CKD development. In contrast, no significant causal relationship was observed between dietary phosphorus and vitamin B2 intake levels and the development of CKD.

Our findings align with prior research. One MR investigation explored the link between phosphorus levels in the bloodstream and CKD in a Chinese cohort [23]. Moreover, this investigation concluded that there is no indication of an association between phosphorus levels in circulation and CKD risk. Genetic variations may play a role in regulating phosphorus levels in the body, managing phosphate concentrations in the bloodstream, and modulating inflammatory responses in kidney tissues [24, 25]. For example, polymorphisms in the FGF23 (fibroblast growth factor 23), KL (Klotho), and PTH (parathyroid hormone) genes have been identified as critical regulators of phosphate homeostasis. The FGF23 gene promotes phosphate excretion via renal tubules and inhibits vitamin D activation [26], while KL polymorphisms can alter the activity of FGF23, influencing phosphate transport and renal function [27]. Variants in the PTH gene affect parathyroid hormone levels, thereby modulating calcium-phosphate metabolism and potentially contributing to CKD pathophysiology [28]. Despite these findings, previous studies have consistently reported no significant association between serum phosphorus levels and CKD risk, suggesting that phosphorus homeostasis alone may not be a direct contributor to CKD development. Instead, it is likely influenced by complex interactions between genetic, metabolic, and environmental factors. These findings highlight the need for further exploration of how genetic variations interact with systemic processes to affect kidney function.

This study demonstrated that there is no causal link between dietary intake of vitamin B2 and the development of CKD. To date, there has been no investigation into the relationship between vitamin B2 and CKD utilizing MR analysis. Vitamin B2 plays a role in nucleic acid and protein metabolism, thereby contributing to cellular energy production. Moreover, it exhibits antioxidant properties, reducing oxidative stress and shielding cells from damage [29, 30]. These biological pathways may elucidate the connection between vitamin B2 intake and CKD incidence. From a genetic perspective, polymorphisms in genes encoding enzymes dependent on riboflavin, such as MTHFR (methylenetetrahydrofolate reductase) and GSS (glutathione synthetase), could influence the efficiency of cellular energy production and antioxidant pathways [31, 32]. For instance, the MTHFR C677 T polymorphism, which affects folate metabolism, is known to alter homocysteine levels, potentially impacting renal function through increased oxidative stress and vascular damage [33]. Similarly, variations in the GSS gene may impair glutathione synthesis, reducing antioxidant defense mechanisms critical for protecting renal tissues from oxidative damage [34]. Based on these findings, further research utilizing genetic factors is warranted to gain a deeper understanding of the relationship between vitamin B2 and kidney function.

This study revealed a weak causal link between genetically predicted levels of vitamin B6 and the risk of CKD development. A previous MR study also supported this finding, demonstrating that higher plasma levels of vitamin B6 were linked to a reduced risk of kidney calculus and CKD, utilizing data from the UK Biobank cohort [35]. Genetic variants associated with vitamin B6 metabolism, such as those near the PSTK and PDXK genes, have been implicated in the regulation of pyridoxal phosphate levels, the active form of vitamin B6 [36, 37]. These genes play a critical role in coenzyme activity and cellular processes related to amino acid metabolism, which may influence kidney function. Biologically, this observation may be attributed to the role of vitamin B6 in decreasing oxalate excretion in urine, thereby potentially lowering the risk of developing calcium oxalate kidney stones [38, 39]. Additionally, SNPs in genes like PDXK, AGXT, and GRHPR can influence oxalate metabolism by regulating gene expression and enzymatic activity [40, 41]. These variations may alter pyridoxal phosphate levels, affect oxalate excretion, and contribute to renal inflammation, thereby impacting individual susceptibility to CKD and kidney stone formation. Further investigation employing genetic factors is essential to gain a deeper understanding of the relationship between vitamin B6 and CKD development.

In addition, the study uncovered a potential weak positive correlation between dietary intake of vitamin C and the development of CKD. To date, no prior research utilizing MR analysis has directly investigated the association between vitamin C and CKD. Previous studies suggest that excessive intake of vitamin D may increase the risk of developing CKD. While vitamin C is known for its antioxidant and anti-inflammatory properties, excessive consumption may promote oxalate formation in the kidneys. This can lead to increased oxalate excretion, thereby elevating the risk of kidney stone formation, and, over time, result in kidney function impairment and progression to CKD [42, 43]. Additionally, high vitamin C intake can enhance iron absorption, potentially causing iron overload in the body. This may increase the production of reactive oxygen species (ROS), leading to oxidative damage to renal tissues [44]. From a genetic perspective, variations in genes involved in vitamin C transport and metabolism, such as SLC23 A1 and SLC23 A2, may influence vitamin C bioavailability and renal clearance [45]. For instance, polymorphisms in SLC23 A1, which encodes a sodium-dependent vitamin C transporter, have been associated with altered plasma and tissue concentrations of vitamin C, potentially modulating its effects on oxalate production and oxidative stress. Similarly, SNPs in genes regulating iron homeostasis, such as HFE and TF, could interact with high vitamin C intake to exacerbate oxidative damage through increased ROS production and iron overload in renal tissues [46, 47]. Therefore, the potential dual effects of vitamin C may vary depending on its intake level, metabolic status, and genetic factors. To better understand these interactions, further in-depth studies are warranted to explore the relationship between vitamin C and CKD. In particular, the use of genetic approaches, such as MR studies, is essential to evaluate the causal associations and provide more robust insights into this relationship.

However, the limitations of our study must be acknowledged. First, despite implementing rigorous quality control measures and utilizing a comprehensive dataset, the potential influence of unmeasured confounding factors on the observed associations cannot be completely disregarded. Second, the findings of this study are associated with variables indicative of a Weak Instrument (WI) in MR analysis, highlighting the importance of careful interpretation. WI occurs when the genetic variation has a minimal effect on the exposure variable. In such situations, the instrument variable has a weak correlation with the actual exposure variable, making the estimates derived using the instrument variable likely to be biased [48].

Third, the SNPs identified in this study as instrumental variables for dietary intake of phosphorus, vitamin B2, B6, and C did not overlap with previously reported genetic loci related to nutrient biomarkers or intake in large-scale GWAS. Notably, the lead SNPs (rs8074317 and rs8074201 in SEPTIN9, rs145928548 in NSUN3, and rs140394939 in PDE4B) have not been previously documented, suggesting the presence of novel or population-specific signals. To examine the biological plausibility of these associations, we reviewed the functional annotations of genes near the identified SNPs. Several loci, such as PDE4B and LRP1B, are involved in metabolic regulation and dietary behavior, indicating potential pathways influencing micronutrient intake [49, 50]. In contrast, well-known nutrient-related loci including SLC23 A1 (vitamin C) and ALPL (vitamin B6) were not detected in our analysis [51, 52]. These discrepancies may reflect differences in genetic architecture and dietary habits across populations, particularly given that our study was conducted in a Korean population, whereas most prior GWAS have focused on European cohorts. Collectively, these findings suggest that the genetic instruments identified in this study may represent East Asian–specific signals and offer novel insights into causal pathways linking micronutrient intake to CKD risk. Further replication in independent and diverse populations is warranted to validate the robustness and generalizability of these associations.

It is essential to proceed with caution as the efficacy of the instrument depends on both the magnitude and precision of the relationship between the genetic instrumental variables (IVs) and the risk factor. Fourth, Pleiotropy, the phenomenon where a single genetic variant influences multiple traits, is a critical consideration in MR studies. Confounding factors, which represent the distortion of causal relationships due to external factors or additional variables, can arise if pleiotropy is present in any form in MR research. Initially, we selected SNPs statistically identified as IVs associated with dietary micronutrient intake. To further ensure the robustness of our instruments, we conducted a comprehensive literature review and mapped phenotype analysis for each SNP. SNPs lacking prior evidence of association with dietary micronutrient intake specifically were categorized as potential pleiotropic SNPs and subsequently excluded from the primary MR analyses. This methodological refinement aimed to reduce pleiotropy and enhance the interpretability and validity of our results. Furthermore, statistical evaluations of pleiotropy were performed using the MR-Egger intercept test and MR-PRESSO global test, neither of which indicated significant horizontal pleiotropy, thus providing additional reassurance regarding the validity of our findings. Despite these precautions, residual pleiotropy cannot be completely ruled out. The IVs retained after this screening process may still have associations with traits such as body mass index (BMI), serum creatinine levels, or other nutrient intakes, potentially affecting our causal inference. Additionally, the typically modest correlation between dietary intake levels and circulating nutrient concentrations raises concerns regarding the biological validity of the selected IVs. Thus, these results should be viewed as indicative rather than definitive. We also emphasize that our findings should be viewed with an understanding of these potential residual pleiotropic effects and biological validity concerns. Future research employing multivariable MR analyses or utilizing biomarker-based instrumental variables is necessary to further validate the observed causal associations and better address remaining methodological challenges.

Finally, to translate the findings of the micronutrients and CKD study into clinical practice, several approaches can be considered. Offering nutritional education emphasizing the importance of micronutrients can assist CKD patients and those at high risk in achieving adequate intake through a balanced diet. Developing treatment and prevention strategies based on observed associations between micronutrients and CKD can be beneficial, especially in cases of identified deficiencies or excesses. Encouraging healthy eating habits related to micronutrient intake can contribute to reducing the risk of CKD development and supporting kidney health maintenance for prevention purposes.

## Conclusion

In conclusion, our study provides suggestive evidence of potential causal relationships between genetically predicted dietary vitamin B6 and vitamin C intake levels and the risk of CKD development. However, given the methodological limitations, including possible residual pleiotropy and modest correlation between dietary intake and circulating nutrient levels, these associations should be interpreted with caution. Despite these limitations, our results offer valuable insights into the potential role of micronutrients in CKD prevention and highlight the need for maintaining balanced nutritional intake.

## Supplementary Information

Supplementary Material 1.

## Data Availability

The K-CHIP consortium genotype data is available upon request under the data sharing policy of National Research Institute of Health, Korea (https://www.koreanchip.org/blank-8). Other data supporting our findings are available from the corresponding author on reasonable request.

## Abbreviations

CKD: Chronic kidney disease
eGFR: Estimate glomerular filtration rate
UACR: Urine albumin to creatinine ratio
MR: Mendelian randomization
GWAS: Genome wide association analysis
MR-PRESSO: Mendelian Randomization Pleiotropy RESidual Sum and Outlier
WI: Weak Instrument
