# Supplementary material for: Mendelian randomization study of micronutrients and development of CKD in a Korean population
Source: Nutr J. 2025 Jun 13;24:90. doi: 10.1186/s12937-025-01160-2 (PMC12166562; doi:10.1186/s12937-025-01160-2)
Supplement: Supplementary file 1 — Supplementary Material 1. [file 12937_2025_1160_MOESM1_ESM.docx]

Supplementary table 1. A total of 62 SNPs associated with dietary phosphorus intake levels from KARE GWAS study

| **CHR** | **Position** | **SNP** | **Nearest gene** | **Alleles** | **MAF** | **BETA** | **SE** | **p-value** |
| --- | --- | --- | --- | --- | --- | --- | --- | --- |
| 17 | 75536480 | rs8074317 | SEPTIN9 | C/T | 0.220 | -0.021 | 0.004 | 6.00E-09 |
| 14 | 41595424 | rs138424249 | LRFN5 | G/A | 0.092 | 0.029 | 0.005 | 6.20E-08 |
| 7 | 90949831 | rs930110 | FZD1 | A/G | 0.530 | 0.015 | 0.003 | 2.50E-07 |
| 2 | 152021778 | rs10193255 | RBM43 | T/C | 0.330 | -0.016 | 0.003 | 4.20E-07 |
| 5 | 143012449 | rs12653682 | HMHB1 | G/T | 0.360 | -0.015 | 0.003 | 9.60E-07 |
| 3 | 157872257 |  | RSRC1 | C/CT | 0.580 | -0.015 | 0.003 | 1.10E-06 |
| 8 | 21761681 | rs77133047 | DOK2 | G/T | 0.180 | -0.019 | 0.004 | 1.10E-06 |
| 9 | 103545759 | rs4742795 | CAVIN4 | C/A | 0.380 | 0.016 | 0.003 | 1.20E-06 |
| 8 | 143115548 | rs4917223 | TSNARE1 | C/T | 0.440 | 0.015 | 0.003 | 1.30E-06 |
| 5 | 172342029 | rs35941893 | ERGIC1 | G/GT | 0.140 | -0.022 | 0.005 | 1.50E-06 |
| 11 | 20544657 | rs7126868 | PRMT3 | A/C | 0.340 | 0.015 | 0.003 | 1.50E-06 |
| 18 | 47474683 | rs78281436 | MYO5B | T/C | 0.046 | -0.036 | 0.008 | 1.50E-06 |
| 1 | 33230670 | rs58302080 | KIAA1522 | C/G | 0.016 | -0.057 | 0.012 | 2.50E-06 |
| 5 | 41019307 | rs145817449 | MROH2B | T/C | 0.018 | -0.053 | 0.011 | 2.70E-06 |
| 11 | 16910814 | rs365215 | PLEKHA7 | A/C | 0.280 | 0.016 | 0.003 | 3.30E-06 |
| 3 | 94996197 | rs145928548 | NSUN3 | G/T | 0.015 | -0.058 | 0.012 | 3.40E-06 |
| 15 | 96917733 |  | NR2F2 | CA/C | 0.964 | -0.038 | 0.008 | 3.60E-06 |
| 3 | 94291570 |  | NSUN3 | A/AT | 0.019 | -0.056 | 0.012 | 3.70E-06 |
| 6 | 92368012 | rs76099546 | MAP3K7 | T/C | 0.079 | -0.026 | 0.006 | 3.90E-06 |
| 22 | 42309993 | rs10708799 | SHISA8 | TG/T | 0.098 | 0.023 | 0.005 | 4.50E-06 |
| 4 | 101501761 | rs2567388 | EMCN | G/T | 0.300 | -0.015 | 0.003 | 4.70E-06 |
| 7 | 150506199 | rs6968855 | TMEM176A | C/T | 0.830 | -0.018 | 0.004 | 5.30E-06 |
| 10 | 124902744 | rs7902081 | HMX3 | T/C | 0.130 | -0.022 | 0.005 | 5.40E-06 |
| 19 | 10064981 | rs11085497 | COL5A3 | G/C | 0.300 | 0.015 | 0.003 | 5.50E-06 |
| 4 | 94292292 | rs184108318 | GRID2 | T/G | 0.016 | 0.060 | 0.013 | 5.90E-06 |
| 11 | 74972225 |  | ARRB1 | CGT/C | 0.480 | 0.015 | 0.003 | 6.60E-06 |
| 3 | 12471871 | rs1152002 | PPARG | C/T | 0.460 | -0.014 | 0.003 | 7.90E-06 |
| 6 | 158438748 | rs144840028 | SYNJ2 | A/G | 0.023 | -0.046 | 0.010 | 9.30E-06 |
| 1 | 226396166 |  | MIXL1 | C/CA | 0.260 | -0.015 | 0.004 | 9.80E-06 |
| 4 | 139786911 | rs62322145 | NOCT | A/G | 0.180 | -0.017 | 0.004 | 9.80E-06 |
| 17 | 67458652 | rs118126226 | MAP2K6 | C/T | 0.016 | -0.052 | 0.012 | 1.10E-05 |
| 20 | 5079882 | rs2093142 | TMEM230 | C/A | 0.780 | -0.017 | 0.004 | 1.10E-05 |
| 2 | 226052785 | rs138511119 | DOCK10 | C/T | 0.019 | -0.052 | 0.012 | 1.20E-05 |
| 8 | 95503360 | rs56069388 | VIRMA | G/A | 0.085 | 0.025 | 0.006 | 1.20E-05 |
| 13 | 93119283 | rs116888405 | GPC5 | G/A | 0.021 | -0.048 | 0.011 | 1.20E-05 |
| 14 | 57979461 | rs577022805 | CCDC198 | C/A | 0.011 | 0.068 | 0.015 | 1.20E-05 |
| 8 | 36582632 |  | KCNU1 | C/CA | 0.110 | 0.021 | 0.005 | 1.30E-05 |
| 1 | 110144024 | rs6691984 | GNAI3 | G/T | 0.240 | -0.015 | 0.004 | 1.40E-05 |
| 5 | 109968039 |  | TMEM232 | G/GA | 0.068 | -0.026 | 0.006 | 1.40E-05 |
| 8 | 50130111 | rs146229093 | PPDPFL | A/G | 0.021 | -0.047 | 0.011 | 1.40E-05 |
| 18 | 38524259 | rs1869282 | PIK3C3 | C/A | 0.300 | 0.014 | 0.003 | 1.40E-05 |
| 19 | 17049245 | rs61133226 | CPAMD8 | G/A | 0.028 | -0.039 | 0.009 | 1.40E-05 |
| 3 | 182904214 | rs11717155 | MCF2L2 | A/T | 0.420 | 0.013 | 0.003 | 1.50E-05 |
| 6 | 2746338 | rs75145876 | MYLK4 | A/T | 0.320 | -0.015 | 0.003 | 1.50E-05 |
| 7 | 90888624 |  | FZD1 | AT/AT | 0.270 | 0.014 | 0.003 | 1.50E-05 |
| 16 | 3467329 | rs2641787 | AC025283.2 | T/G | 0.931 | -0.026 | 0.006 | 1.50E-05 |
| 7 | 88105733 | rs74914709 | STEAP4 | C/T | 0.013 | 0.063 | 0.015 | 1.60E-05 |
| 7 | 90982131 | rs62470167 | FZD1 | A/G | 0.280 | 0.014 | 0.003 | 1.60E-05 |
| 10 | 36656814 | rs17544598 | FZD8 | A/G | 0.039 | 0.033 | 0.008 | 1.60E-05 |
| 17 | 5166504 | rs187412047 | RABEP1 | C/T | 0.018 | 0.049 | 0.011 | 1.60E-05 |
| 8 | 80613324 | rs2467802 | STMN2 | A/G | 0.120 | -0.019 | 0.005 | 1.70E-05 |
| 3 | 80284713 | rs1343463419 | ROBO1 | T/A | 0.013 | -0.058 | 0.013 | 1.80E-05 |
| 20 | 9542145 | rs4053116 | PAK5 | A/C | 0.290 | 0.014 | 0.003 | 1.80E-05 |
| 2 | 163476673 | rs55967009 | KCNH7 | G/T | 0.400 | 0.013 | 0.003 | 1.90E-05 |
| 3 | 2924686 | rs4685575 | CNTN4 | G/A | 0.770 | -0.015 | 0.004 | 1.90E-05 |
| 5 | 128058227 | rs144797062 | SLC27A6 | T/A | 0.015 | -0.054 | 0.013 | 1.90E-05 |
| 6 | 87647368 | rs9362377 | HTR1E | G/A | 0.016 | -0.052 | 0.012 | 1.90E-05 |
| 6 | 139429505 |  | HECA | C/CA | 0.014 | -0.059 | 0.014 | 1.90E-05 |
| 9 | 31093665 | rs144408828 | ACO1 | AGTC/A | 0.240 | -0.015 | 0.004 | 1.90E-05 |
| 11 | 75713515 | rs145067629 | UVRAG | C/A | 0.032 | -0.037 | 0.009 | 1.90E-05 |
| 15 | 98300354 | rs12911239 | ARRDC4 | T/C | 0.500 | -0.013 | 0.003 | 1.90E-05 |
| 2 | 199427779 | rs1598350 | PLCL1 | G/T | 0.979 | 0.045 | 0.011 | 2.00E-05 |

KARE, Korean association resource; SNP, single nucleotide polymorphism; MAF, minor allele frequency; SE, standard error;

Supplementary table 2. A total of 56 SNPs associated with dietary vitamin B2 intake levels from KARE GWAS study

| **CHR** | **Position** | **SNP** | **Nearest gene** | **Alleles** | **MAF** | **BETA** | **SE** | **p-value** |
| --- | --- | --- | --- | --- | --- | --- | --- | --- |
| 19 | 10080619 |  | COL5A3 | A/G | 0.550 | 0.020 | 0.004 | 1.50E-08 |
| 17 | 75536469 | rs8074201 | SEPTIN9 | C/T | 0.220 | -0.024 | 0.004 | 3.90E-08 |
| 6 | 135437769 | rs78791991 | HBS1L | T/C | 0.035 | 0.049 | 0.010 | 5.20E-07 |
| 1 | 43917601 | rs111686948 | HYI,SZT2 | C/T | 0.020 | 0.063 | 0.013 | 6.80E-07 |
| 1 | 1980190 | rs147957210 | PRKCZ | T/G | 0.032 | -0.048 | 0.010 | 1.60E-06 |
| 16 | 49213050 | rs192749599 | CBLN1 | A/G | 0.025 | 0.054 | 0.011 | 1.70E-06 |
| 5 | 18048203 | rs12517936 | H3Y1 | C/T | 0.440 | -0.017 | 0.004 | 2.30E-06 |
| 2 | 52618835 | rs2727883 | ASB3 | A/T | 0.610 | 0.018 | 0.004 | 2.60E-06 |
| 13 | 45490877 | rs141095648 | NUFIP1 | C/A | 0.075 | -0.032 | 0.007 | 2.80E-06 |
| 3 | 26687577 | rs17018468 | LRRC3B | A/G | 0.290 | -0.018 | 0.004 | 3.20E-06 |
| 8 | 21759493 | rs11785978 | DOK2 | G/C | 0.210 | -0.020 | 0.004 | 3.20E-06 |
| 5 | 49779936 | rs1974852 | EMB | C/A | 0.170 | -0.023 | 0.005 | 3.40E-06 |
| 3 | 160966816 | rs80277692 | NMD3 | A/G | 0.140 | -0.024 | 0.005 | 3.60E-06 |
| 1 | 183416929 | rs144877614 | SMG7 | G/A | 0.021 | -0.058 | 0.012 | 3.70E-06 |
| 3 | 158106411 | rs200008658 | RSRC1 | T/A | 0.300 | 0.018 | 0.004 | 4.10E-06 |
| 2 | 163442456 | rs1485984 | KCNH7 | C/T | 0.380 | 0.017 | 0.004 | 4.20E-06 |
| 10 | 97124303 | rs72822548 | SORBS1 | G/A | 0.370 | -0.017 | 0.004 | 4.30E-06 |
| 17 | 70213909 | rs4793278 | SOX9 | C/G | 0.380 | -0.016 | 0.004 | 5.30E-06 |
| 6 | 14995772 | rs6905335 | JARID2 | A/G | 0.370 | -0.017 | 0.004 | 5.40E-06 |
| 20 | 5079882 | rs2093142 | TMEM230 | C/A | 0.780 | -0.021 | 0.005 | 5.50E-06 |
| 1 | 236908689 | rs533518931 | ACTN2 | G/C | 0.013 | 0.075 | 0.017 | 5.70E-06 |
| 2 | 152021778 | rs10193255 | RBM43 | T/C | 0.330 | -0.017 | 0.004 | 6.40E-06 |
| 14 | 41595424 | rs138424249 | LRFN5 | G/A | 0.092 | 0.029 | 0.007 | 6.60E-06 |
| 21 | 18958719 |  | CXADR | GTA/GTA | 0.410 | 0.016 | 0.004 | 7.10E-06 |
| 7 | 90957471 | rs1346667 | FZD1 | A/G | 0.200 | 0.020 | 0.005 | 7.20E-06 |
| 7 | 150506199 | rs6968855 | TMEM176A | C/T | 0.830 | -0.021 | 0.005 | 7.30E-06 |
| 4 | 90547074 |  | SNCA | C/T | 0.350 | -0.017 | 0.004 | 7.50E-06 |
| 1 | 4064104 | rs10915570 | C1orf174 | C/T | 0.670 | 0.017 | 0.004 | 7.60E-06 |
| 19 | 47081250 | rs11670297 | CALM3 | G/A | 0.400 | 0.016 | 0.004 | 7.60E-06 |
| 1 | 183584284 | rs548054457 | ARPC5 | C/T | 0.022 | 0.058 | 0.013 | 7.70E-06 |
| 5 | 41019307 | rs145817449 | MROH2B | T/C | 0.018 | -0.060 | 0.013 | 8.00E-06 |
| 7 | 141798110 | rs10215743 | MGAM | G/A | 0.680 | 0.018 | 0.004 | 8.50E-06 |
| 5 | 111168720 | rs7703429 | NREP | A/G | 0.030 | 0.046 | 0.010 | 8.60E-06 |
| 4 | 153150493 | rs185500746 | FBXW7 | T/C | 0.024 | -0.051 | 0.011 | 8.70E-06 |
| 5 | 4579173 |  | ADAMTS16 | CTA/CTA | 0.400 | 0.017 | 0.004 | 8.70E-06 |
| 9 | 38203435 | rs10973705 | SHB | C/T | 0.470 | -0.016 | 0.004 | 8.70E-06 |
| 16 | 17650641 | rs8049297 | XYLT1 | T/C | 0.056 | 0.038 | 0.009 | 8.80E-06 |
| 17 | 18271681 | rs182654382 | SHMT1 | A/G | 0.046 | 0.041 | 0.009 | 9.10E-06 |
| 11 | 17589673 | rs7131109 | OTOG | C/A | 0.270 | -0.019 | 0.004 | 9.20E-06 |
| 6 | 40388410 | rs146438270 | LRFN2 | C/T | 0.020 | -0.061 | 0.014 | 9.60E-06 |
| 22 | 50883047 |  | PPP6R2 | G/G | 0.470 | 0.016 | 0.004 | 9.60E-06 |
| 1 | 11791387 | rs59461005 | AGTRAP | G/A | 0.039 | -0.042 | 0.010 | 9.70E-06 |
| 2 | 43241704 | rs76568400 | ZFP36L2 | A/G | 0.020 | -0.057 | 0.013 | 1.00E-05 |
| 14 | 58216676 | rs117366406 | SLC35F4 | G/C | 0.011 | 0.081 | 0.018 | 1.00E-05 |
| 5 | 155593021 | rs116875571 | SGCD | A/G | 0.052 | -0.035 | 0.008 | 1.10E-05 |
| 6 | 158438748 | rs144840028 | SYNJ2 | A/G | 0.023 | -0.054 | 0.012 | 1.10E-05 |

KARE, Korean association resource; SNP, single nucleotide polymorphism; MAF, minor allele frequency; SE, standard error;

Supplementary table 3. A total of 66 SNPs associated with dietary vitamin B6 levels from KARE GWAS study

| **CHR** | **Position** | **SNP** | **Nearest gene** | **Alleles** | **MAF** | **BETA** | **SE** | **p-value** |
| --- | --- | --- | --- | --- | --- | --- | --- | --- |
| 3 | 94996197 | rs145928548 | NSUN3 | G/T | 0.015 | -0.072 | 0.014 | 9.20E-08 |
| 5 | 143104177 | rs184559817 | HMHB1 | G/A | 0.010 | 0.088 | 0.017 | 1.50E-07 |
| 19 | 30547701 | rs138959558 | URI1 | A/G | 0.028 | 0.054 | 0.011 | 3.80E-07 |
| 3 | 94454752 | rs148237512 | NSUN3 | C/T | 0.015 | -0.069 | 0.014 | 5.30E-07 |
| 19 | 36346318 | rs77055181 | NPHS1 | G/A | 0.086 | -0.029 | 0.006 | 7.10E-07 |
| 5 | 172342029 | rs35941893 | ERGIC1 | G/GT | 0.140 | -0.024 | 0.005 | 8.20E-07 |
| 7 | 25636379 | rs2813894 | NPVF | G/A | 0.850 | 0.022 | 0.005 | 8.40E-07 |
| 9 | 103545759 | rs4742795 | CAVIN4 | C/A | 0.380 | 0.017 | 0.004 | 8.90E-07 |
| 16 | 57137533 | rs12600010 | CPNE2 | C/G | 0.200 | -0.020 | 0.004 | 1.40E-06 |
| 5 | 111125127 | rs931684 | NREP | A/G | 0.290 | -0.017 | 0.004 | 1.80E-06 |
| 10 | 127811449 | rs141302176 | ADAM12 | G/A | 0.026 | -0.048 | 0.010 | 2.10E-06 |
| 17 | 75533220 | rs116628291 | SEPTIN9 | G/C | 0.230 | -0.019 | 0.004 | 2.10E-06 |
| 6 | 86071012 | rs77331108 | NT5E | A/G | 0.017 | 0.059 | 0.013 | 2.40E-06 |
| 4 | 139786911 | rs62322145 | NOCT | A/G | 0.180 | -0.020 | 0.004 | 4.10E-06 |
| 6 | 21687571 | rs1973986 | SOX4 | T/A | 0.850 | -0.021 | 0.005 | 4.20E-06 |
| 1 | 7834026 | rs2071987 | VAMP3 | G/A | 0.390 | 0.015 | 0.003 | 5.60E-06 |
| 3 | 182902711 |  | MCF2L2 | CT/C | 0.430 | 0.015 | 0.003 | 5.90E-06 |
| 8 | 5310952 | rs184679994 | CSMD1 | G | 0.010 | -0.078 | 0.017 | 6.90E-06 |
| 7 | 6602874 |  | GRID2IP | C/CA | 0.250 | 0.018 | 0.004 | 7.00E-06 |
| 8 | 21759493 | rs11785978 | DOK2 | G/C | 0.210 | -0.018 | 0.004 | 7.30E-06 |
| 1 | 203722880 | rs375382680 | ATP2B4 | C/T | 0.020 | -0.052 | 0.012 | 7.40E-06 |
| 6 | 170517448 | rs12192239 | DLL1 | C/T | 0.630 | -0.016 | 0.004 | 7.90E-06 |
| 2 | 71246505 | rs35280662 | AC007040.2 | G/A | 0.035 | -0.042 | 0.009 | 8.10E-06 |
| 6 | 92368012 | rs76099546 | MAP3K7 | T/C | 0.079 | -0.027 | 0.006 | 8.10E-06 |
| 2 | 52559485 | rs2727890 | NRXN1 | T/A | 0.560 | 0.015 | 0.003 | 8.70E-06 |
| 11 | 11231079 | rs60577634 | GALNT18 | G/GC | 0.160 | 0.021 | 0.005 | 9.40E-06 |
| 3 | 149732830 | rs9289799 | PFN2 | A/G | 0.200 | 0.018 | 0.004 | 9.50E-06 |
| 6 | 149550529 |  | TAB2 | CGT/C | 0.740 | -0.017 | 0.004 | 1.00E-05 |
| 2 | 67915257 | rs118150927 | ETAA1 | A/C | 0.130 | 0.021 | 0.005 | 1.10E-05 |
| 4 | 53562564 |  | ERVMER34-1 | CA/C | 0.210 | -0.018 | 0.004 | 1.10E-05 |
| 4 | 163756013 | rs79094951 | NAF1 | C/G | 0.028 | 0.043 | 0.010 | 1.10E-05 |
| 6 | 111644665 | rs240966 | MFSD4B,REV3L | A/G | 0.790 | -0.018 | 0.004 | 1.10E-05 |
| 9 | 112073430 | rs7357791 | EPB41L4B | T/C | 0.056 | 0.031 | 0.007 | 1.10E-05 |
| 1 | 226396166 |  | MIXL1 | C/CA | 0.260 | -0.017 | 0.004 | 1.20E-05 |
| 1 | 245494103 |  | KIF26B | G/GATC | 0.180 | -0.020 | 0.005 | 1.20E-05 |
| 2 | 100507688 | rs78350324 | AFF3 | T/C | 0.074 | 0.028 | 0.006 | 1.20E-05 |
| 5 | 49846117 | rs16882482 | EMB | C/G | 0.084 | 0.026 | 0.006 | 1.20E-05 |
| 6 | 42588238 | rs9357399 | UBR2 | G/T | 0.760 | -0.017 | 0.004 | 1.20E-05 |
| 6 | 164223344 |  | QKI | C/CA | 0.690 | 0.016 | 0.004 | 1.20E-05 |
| 11 | 84306528 |  | DLG2 | T/TA | 0.120 | -0.023 | 0.005 | 1.20E-05 |
| 5 | 105884811 | rs187697531 | EFNA5 | G/A | 0.017 | 0.060 | 0.014 | 1.30E-05 |
| 9 | 89974633 | rs11141767 | DAPK1 | A/G | 0.110 | -0.023 | 0.005 | 1.30E-05 |
| 12 | 51963994 | rs372267467 | SCN8A | T/A | 0.130 | -0.023 | 0.005 | 1.30E-05 |
| 1 | 33306114 | rs552451551 | S100PBP | C/T | 0.011 | -0.074 | 0.017 | 1.40E-05 |
| 6 | 132380078 | rs9321322 | CCN2 | A/G | 0.780 | -0.018 | 0.004 | 1.40E-05 |
| 13 | 85780099 | rs146128635 | SLITRK6 | C/T | 0.011 | -0.071 | 0.016 | 1.40E-05 |
| 15 | 98816793 | rs12442666 | ARRDC4 | T/C | 0.380 | 0.015 | 0.003 | 1.40E-05 |
| 4 | 101501761 | rs2567388 | EMCN | G/T | 0.300 | -0.015 | 0.004 | 1.50E-05 |
| 4 | 144516815 | rs6812104 | FREM3 | C/T | 0.360 | 0.016 | 0.004 | 1.50E-05 |
| 11 | 2671096 | rs201434624 | KCNQ1 | A/G | 0.012 | 0.070 | 0.016 | 1.50E-05 |
| 19 | 12732673 | rs112829751 | ZNF490,ZNF791 | T/C | 0.063 | 0.030 | 0.007 | 1.60E-05 |
| 20 | 18824142 | rs13037731 | SCP2D1 | C/T | 0.082 | 0.026 | 0.006 | 1.60E-05 |
| 1 | 2382334 | rs143569985 | PLCH2 | GT/G | 0.028 | -0.044 | 0.010 | 1.70E-05 |
| 17 | 464044 | rs140874269 | VPS53 | C/T | 0.020 | 0.050 | 0.012 | 1.70E-05 |
| 5 | 79298349 | rs437605 | THBS4 | C/T | 0.360 | 0.014 | 0.003 | 1.80E-05 |
| 9 | 18193796 | rs2187455 | ADAMTSL1 | G/A | 0.250 | 0.016 | 0.004 | 1.90E-05 |
| 10 | 97125360 | rs10882585 | SORBS1 | G/C | 0.310 | -0.015 | 0.004 | 1.90E-05 |
| 13 | 102247971 | rs9554809 | ITGBL1 | G/A | 0.710 | -0.015 | 0.004 | 1.90E-05 |
| 16 | 78274211 |  | WWOX | TA/T | 0.100 | -0.023 | 0.006 | 1.90E-05 |
| 18 | 68123131 | rs141217439 | SOCS6 | G/A | 0.016 | -0.055 | 0.013 | 1.90E-05 |
| 4 | 14195044 | rs16889812 | BOD1L1 | T/C | 0.180 | 0.018 | 0.004 | 2.00E-05 |
| 11 | 23572099 |  | CCDC179 | G/GT | 0.160 | 0.020 | 0.005 | 2.00E-05 |
| 8 | 49860653 | rs147902155 | SNAI2 | G/A | 0.029 | -0.042 | 0.010 | 2.10E-05 |
| 11 | 116456275 | rs117487083 | BUD13 | T/C | 0.026 | -0.043 | 0.010 | 2.10E-05 |
| 12 | 32215808 | rs56374038 | BICD1 | C/T | 0.022 | 0.048 | 0.011 | 2.10E-05 |
| 13 | 50751422 | rs1241067 | KCNRG | C/T | 0.850 | -0.019 | 0.005 | 2.10E-05 |

KARE, Korean association resource; SNP, single nucleotide polymorphism; MAF, minor allele frequency; SE, standard error;

Supplementary table 4. A total of 50 SNPs associated with dietary vitamin C intake levels from KARE GWAS study

| **CHR** | **Position** | **SNP** | **Nearest gene** | **Alleles** | **MAF** | **BETA** | **SE** | **p-value** |
| --- | --- | --- | --- | --- | --- | --- | --- | --- |
| 2 | 123756834 | rs71398097 | CNTNAP5 | GT/G | 0.072 | -0.049 | 0.010 | 1.90E-07 |
| 2 | 162716138 | rs188527347 | SLC4A10 | T/C | 0.036 | 0.064 | 0.013 | 7.30E-07 |
| 1 | 66289238 | rs140394939 | PDE4B | C/A | 0.015 | -0.098 | 0.020 | 1.20E-06 |
| 2 | 142216788 | rs16846116 | LRP1B | G/A | 0.070 | 0.044 | 0.009 | 1.20E-06 |
| 11 | 84219443 |  | DLG2 | CT/C | 0.170 | -0.034 | 0.007 | 1.40E-06 |
| 14 | 92485304 |  | TRIP11 | C/CA | 0.020 | 0.086 | 0.018 | 1.50E-06 |
| 6 | 111874889 |  | TRAF3IP2 | C/CA | 0.360 | -0.024 | 0.005 | 1.80E-06 |
| 8 | 49860653 | rs147902155 | SNAI2 | G/A | 0.029 | -0.068 | 0.014 | 2.00E-06 |
| 5 | 76879013 | rs57586676 | WDR41 | A/AT | 0.140 | -0.033 | 0.007 | 2.50E-06 |
| 1 | 22291655 |  | CELA3B | AT/A | 0.570 | -0.023 | 0.005 | 2.80E-06 |
| 1 | 96785080 | rs12031723 | PTBP2 | T/C | 0.250 | -0.026 | 0.006 | 2.90E-06 |
| 6 | 65499777 |  | EYS | GAT/G | 0.680 | 0.024 | 0.005 | 3.60E-06 |
| 9 | 2853549 | rs79325616 | PUM3 | A/T | 0.061 | 0.046 | 0.010 | 3.90E-06 |
| 8 | 62649094 | rs11785234 | ASPH | C/T | 0.130 | -0.032 | 0.007 | 4.30E-06 |
| 5 | 141003520 | rs76463900 | HDAC3 | G/A | 0.140 | 0.032 | 0.007 | 4.60E-06 |
| 7 | 116428700 | rs117585786 | MET | A/G | 0.012 | -0.098 | 0.022 | 5.40E-06 |
| 5 | 35245852 | rs10074128 | PRLR | A/G | 0.390 | 0.022 | 0.005 | 6.90E-06 |
| 3 | 79054859 | rs144833267 | ROBO1 | C/T | 0.025 | 0.073 | 0.016 | 7.20E-06 |
| 7 | 25610862 | rs2521745 | NPVF | G/A | 0.800 | 0.026 | 0.006 | 7.30E-06 |
| 3 | 154911267 | rs149736051 | MME | TGCA/T | 0.089 | 0.037 | 0.008 | 7.40E-06 |
| 2 | 218818468 | rs3828277 | TNS1 | C/T | 0.065 | -0.042 | 0.009 | 7.50E-06 |
| 8 | 96364665 | rs146782291 | C8orf37 | G/A | 0.044 | 0.052 | 0.012 | 8.90E-06 |
| 5 | 51224218 | rs75461325 | ISL1 | AT/A | 0.029 | 0.064 | 0.015 | 9.30E-06 |
| 1 | 226670999 | rs898833 | STUM | C/T | 0.230 | -0.024 | 0.006 | 9.40E-06 |
| 1 | 74576998 | rs140541944 | LRRIQ3 | G/A | 0.016 | 0.089 | 0.020 | 1.10E-05 |
| 7 | 821588 | rs13307964 | DNAAF5 | C/T | 0.075 | 0.040 | 0.009 | 1.10E-05 |
| 12 | 22989382 | rs2466887 | ETNK1 | C/T | 0.360 | -0.023 | 0.005 | 1.10E-05 |
| 2 | 162215872 | rs74564505 | PSMD14 | A/G | 0.022 | 0.075 | 0.017 | 1.20E-05 |
| 13 | 42049481 | rs575233021 | RGCC | T/C | 0.017 | -0.087 | 0.020 | 1.30E-05 |
| 4 | 38369349 | rs138058868 | TBC1D1 | A/T | 0.058 | 0.043 | 0.010 | 1.40E-05 |
| 10 | 117074637 | rs150633355 | ATRNL1 | CA/C | 0.051 | 0.047 | 0.011 | 1.40E-05 |
| 15 | 44390736 |  | FRMD5 | A/AG | 0.490 | 0.021 | 0.005 | 1.40E-05 |
| 15 | 65140715 | rs35048171 | AC069368.1,PLEKHO2 | C/G | 0.120 | -0.031 | 0.007 | 1.40E-05 |
| 16 | 49252034 | rs59884489 | CBLN1 | C/T | 0.190 | -0.026 | 0.006 | 1.40E-05 |
| 1 | 170949229 | rs2206565 | MROH9 | G/C | 0.370 | 0.021 | 0.005 | 1.70E-05 |
| 1 | 7864933 |  | PER3 | C/CT | 0.390 | -0.021 | 0.005 | 1.80E-05 |
| 1 | 46105725 |  | GPBP1L1 | G/GA | 0.230 | 0.025 | 0.006 | 1.80E-05 |
| 10 | 118108501 | rs12572549 | CCDC172 | A/G | 0.110 | 0.034 | 0.008 | 1.80E-05 |
| 12 | 51963994 | rs372267467 | SCN8A | T/A | 0.130 | -0.032 | 0.007 | 1.80E-05 |
| 10 | 92279911 |  | HTR7 | T/TG | 0.620 | 0.021 | 0.005 | 1.90E-05 |
| 12 | 20550206 | rs10841505 | PDE3A | C/G | 0.500 | -0.020 | 0.005 | 1.90E-05 |
| 18 | 67176602 | rs9319782 | DOK6 | G/A | 0.660 | -0.023 | 0.005 | 1.90E-05 |
| 20 | 5635153 | rs11466861 | GPCPD1 | TGA/T | 0.260 | -0.023 | 0.005 | 1.90E-05 |
| 5 | 132242490 | rs143942483 | AFF4 | TG/T | 0.120 | 0.030 | 0.007 | 2.00E-05 |
| 4 | 38575616 | rs72150215 | KLF3 | AT/A | 0.270 | 0.023 | 0.005 | 2.10E-05 |
| 8 | 90183763 | rs77145476 | RIPK2 | T/C | 0.065 | 0.040 | 0.010 | 2.10E-05 |
| 11 | 20378124 | rs11025523 | HTATIP2 | G/A | 0.150 | 0.028 | 0.007 | 2.10E-05 |
| 17 | 79894500 | rs2272031 | PYCR1 | T/C | 0.016 | 0.078 | 0.018 | 2.10E-05 |
| 20 | 9543688 | rs2297347 | PAK5 | T/G | 0.290 | 0.022 | 0.005 | 2.10E-05 |
| 21 | 34382450 | rs8131801 | OLIG2 | G/C | 0.340 | -0.022 | 0.005 | 2.10E-05 |

KARE, Korean association resource; SNP, single nucleotide polymorphism; MAF, minor allele frequency; SE, standard error;

Supplementary table 5. Associations of individual genetic instruments for dietary phosphorus intake levels with CKD development

| **CHR** | **SNP** | **Nearest Genes** | **Mapped phenotypes** | **Effect allele** | **MAF** | **Dietary phosphorus intake** | | | **CKD** | | |
| --- | --- | --- | --- | --- | --- | --- | --- | --- | --- | --- | --- |
|  |  |  |  |  |  | **Beta** | **SE** | **P-value** | **Beta** | **SE** | **P-value** |
| 17 | rs8074317 | SEPTIN9 | Phosphorus, vitamin B2 intake | T | 0.220 | -0.021 | 0.0036 | 6.00E-09 | -0.024 | 0.099 | 0.806 |
| 14 | rs138424249 | LRFN5 | Phosphorus Intake | A | 0.092 | 0.029 | 0.0054 | 6.20E-08 | -0.179 | 0.145 | 0.218 |
| 7 | rs930110 | FZD1 | Phosphorus Intake | G | 0.530 | 0.015 | 0.003 | 2.50E-07 | 0.019 | 0.103 | 0.850 |
| 2 | rs10193255 | RBM43 | Phosphorus Intake | C | 0.330 | -0.016 | 0.0032 | 4.20E-07 | 0.032 | 0.084 | 0.700 |
| 8 | rs77133047 | DOK2 | Phosphorus Intake | T | 0.180 | -0.019 | 0.0039 | 1.10E-06 | -0.153 | 0.129 | 0.234 |
| 9 | rs4742795 | CAVIN4 | Gastric cancer | A | 0.380 | 0.016 | 0.0032 | 1.20E-06 | -0.091 | 0.084 | 0.279 |
| 8 | rs4917223 | TSNARE1 | WC, TC, Creatinine | T | 0.440 | 0.015 | 0.003 | 1.30E-06 | -0.162 | 0.099 | 0.101 |
| 11 | rs7126868 | PRMT3 | Phosphorus Intake | C | 0.340 | 0.015 | 0.0032 | 1.50E-06 | 0.014 | 0.101 | 0.889 |
| 18 | rs78281436 | MYO5B | Phosphorus Intake, HDL | C | 0.046 | -0.036 | 0.0075 | 1.50E-06 | 0.202 | 0.215 | 0.347 |
| 5 | rs145817449 | MROH2B | Phosphorus Intake, Gastric cancer | C | 0.018 | -0.053 | 0.011 | 2.70E-06 | -0.307 | 0.280 | 0.272 |
| 11 | rs365215 | PLEKHA7 | Phosphorus Intake, BMI | C | 0.280 | 0.016 | 0.0034 | 3.30E-06 | 0.090 | 0.104 | 0.385 |
| 3 | rs145928548 | NSUN3 | Weight, BMI, Sodium intake | T | 0.015 | -0.058 | 0.012 | 3.40E-06 | 0.319 | 0.351 | 0.363 |
| 6 | rs76099546 | MAP3K7 | Thyroid cancer | C | 0.079 | -0.026 | 0.0056 | 3.90E-06 | -0.166 | 0.176 | 0.347 |
| 4 | rs2567388 | EMCN | Phosphorus Intake, hyperlipidemia | T | 0.300 | -0.015 | 0.0033 | 4.70E-06 | -0.067 | 0.084 | 0.427 |
| 7 | rs6968855 | TMEM176A | Height, Albumin, cholesterol intake | T | 0.830 | -0.018 | 0.004 | 5.30E-06 | 0.084 | 0.135 | 0.536 |
| 10 | rs7902081 | HMX3 | Phosphorus Intake | C | 0.130 | -0.022 | 0.0049 | 5.40E-06 | -0.070 | 0.131 | 0.595 |
| 4 | rs184108318 | GRID2 | Weight, BMI | G | 0.016 | 0.06 | 0.013 | 5.90E-06 | 0.237 | 0.311 | 0.447 |
| 3 | rs1152002 | PPARG | HDL, LDL, DM | T | 0.460 | -0.014 | 0.0032 | 7.90E-06 | -0.074 | 0.079 | 0.349 |
| 4 | rs62322145 | NOCT | height, weight | G | 0.180 | -0.017 | 0.0039 | 9.80E-06 | -0.125 | 0.103 | 0.227 |

KARE, Korea Association resource; SNP, single nucleotide polymorphism; MAF, minor allele frequency; SE, standard error; WC, Waist circumference; TC, Total cholesterol; HDL, High-Density Lipoprotein; BMI, Body mass index; DM, Diabetes mellitus; LDL, Low-Density Lipoprotein;

Supplementary table 6. Associations of individual genetic instruments for dietary vitamin B2 intake levels with CKD development

| **CHR** | **SNP** | **Nearest Genes** | **Mapped phenotypes** | **Effect allele** | **MAF** | **Dietary vitamin B2 intake** | | | **CKD** | | |
| --- | --- | --- | --- | --- | --- | --- | --- | --- | --- | --- | --- |
|  |  |  |  |  |  | **Beta** | **SE** | **P-value** | **Beta** | **SE** | **P-value** |
| 17 | rs8074201 | SEPTIN9 | Vitamin B2, phosphorus intake | T | 0.220 | -0.024 | 0.004 | 3.90E-08 | -0.024 | 0.099 | 0.804 |
| 1 | rs147957210 | PRKCZ | Vitamin B2 intake, BMI | G | 0.032 | -0.048 | 0.010 | 1.60E-06 | -0.349 | 0.239 | 0.144 |
| 5 | rs12517936 | H3Y1 | Alanine Aminotransferase | T | 0.440 | -0.017 | 0.004 | 2.30E-06 | -0.030 | 0.081 | 0.716 |
| 13 | rs141095648 | NUFIP1 | Vitamin B2, sodium intake | A | 0.075 | -0.032 | 0.007 | 2.80E-06 | 0.004 | 0.156 | 0.980 |
| 3 | rs17018468 | LRRC3B | Vitamin B2, iron, calcium intake | G | 0.290 | -0.018 | 0.004 | 3.20E-06 | -0.037 | 0.087 | 0.667 |
| 5 | rs1974852 | EMB | Vitamin B2 intake, DM | A | 0.170 | -0.023 | 0.005 | 3.40E-06 | 0.008 | 0.110 | 0.940 |
| 3 | rs80277692 | NMD3 | Vitamin B2 intake, Gastric cancer | G | 0.140 | -0.024 | 0.005 | 3.60E-06 | -0.104 | 0.117 | 0.373 |
| 2 | rs1485984 | KCNH7 | Vitamin B2 intake, Creatinine | T | 0.380 | 0.017 | 0.004 | 4.20E-06 | 0.074 | 0.082 | 0.368 |
| 10 | rs72822548 | SORBS1 | Vitamin B2 intake, RBC | A | 0.370 | -0.017 | 0.004 | 4.30E-06 | -0.116 | 0.085 | 0.174 |
| 6 | rs6905335 | JARID2 | Triglycerides, calcium intake | G | 0.370 | -0.017 | 0.004 | 5.40E-06 | -0.052 | 0.095 | 0.586 |
| 2 | rs10193255 | RBM43 | Vitamin B2, phosphorus intake | C | 0.330 | -0.017 | 0.004 | 6.40E-06 | 0.032 | 0.084 | 0.700 |
| 7 | rs1346667 | FZD1 | Phosphorus, vitamin B2, calcium intake | G | 0.200 | 0.020 | 0.005 | 7.20E-06 | 0.029 | 0.128 | 0.820 |
| 1 | rs10915570 | C1orf174 | Platelet, thyroid cancer | T | 0.670 | 0.017 | 0.004 | 7.60E-06 | 0.026 | 0.092 | 0.781 |
| 5 | rs145817449 | MROH2B | Gastric cancer, calcium, phosphorus intake | C | 0.018 | -0.060 | 0.013 | 8.00E-06 | -0.307 | 0.280 | 0.272 |
| 5 | rs7703429 | NREP | Platelet, RBC, Weight | G | 0.030 | 0.046 | 0.010 | 8.60E-06 | 0.130 | 0.220 | 0.554 |
| 9 | rs10973705 | SHB | Vitamin B2, Calcium intake | T | 0.470 | -0.016 | 0.004 | 8.70E-06 | -0.133 | 0.081 | 0.100 |
| 4 | rs185500746 | FBXW7 | Vitamin B2 intake, DM | C | 0.024 | -0.051 | 0.011 | 8.70E-06 | -0.080 | 0.253 | 0.750 |
| 16 | rs8049297 | XYLT1 | Height, breast cancer | C | 0.056 | 0.038 | 0.009 | 8.80E-06 | 0.113 | 0.233 | 0.628 |
| 6 | rs146438270 | LRFN2 | Vitamin B2 intake, BMI | T | 0.020 | -0.061 | 0.014 | 9.60E-06 | 0.038 | 0.358 | 0.915 |

KARE, Korea Association resource; SNP, single nucleotide polymorphism; MAF, minor allele frequency; SE, standard error; RBC, red blood cell; BMI, Body mass index; DM, Diabetes mellitus;

Supplementary table 7. Associations of individual genetic instruments for dietary vitamin B6 intake levels with CKD development

| **CHR** | **SNP** | **Nearest Genes** | **Mapped phenotypes** | **Effect allele** | **MAF** | **Dietary vitamin b6 intake** | | | **CKD** | | |
| --- | --- | --- | --- | --- | --- | --- | --- | --- | --- | --- | --- |
|  |  |  |  |  |  | **Beta** | **SE** | **P-value** | **Beta** | **SE** | **P-value** |
| 3 | rs145928548 | NSUN3 | Vitamin B6 intake | T | 0.015 | -0.072 | 0.014 | 9.20E-08 | 0.319 | 0.351 | 0.363 |
| 5 | rs184559817 | HMHB1 | Vitamin B6 intake, Height | A | 0.010 | 0.088 | 0.017 | 1.50E-07 | -0.421 | 0.472 | 0.372 |
| 3 | rs148237512 | NSUN3 | Vitamin B6 intake | T | 0.015 | -0.069 | 0.014 | 5.30E-07 | 0.450 | 0.369 | 0.223 |
| 19 | rs77055181 | NPHS1 | Vitamin B6, Iron intake, Creatinine | A | 0.086 | -0.029 | 0.006 | 7.10E-07 | 0.027 | 0.160 | 0.867 |
| 9 | rs4742795 | CAVIN4 | Vitamin B6, Niacin intake | A | 0.380 | 0.017 | 0.004 | 8.90E-07 | -0.091 | 0.084 | 0.279 |
| 5 | rs931684 | NREP | Vitamin B6, Platelet | G | 0.290 | -0.017 | 0.004 | 1.80E-06 | 0.019 | 0.091 | 0.837 |
| 10 | rs141302176 | ADAM12 | Vitamin B6, Niacin, Iron intake | A | 0.026 | -0.048 | 0.010 | 2.10E-06 | 0.169 | 0.243 | 0.486 |
| 1 | rs2071987 | VAMP3 | Vitamin B6, Iron, Folate intake | A | 0.390 | 0.015 | 0.003 | 5.60E-06 | -0.043 | 0.087 | 0.623 |
| 1 | rs375382680 | ATP2B4 | Vitamin B6, Fiber, Iron intake | T | 0.020 | -0.052 | 0.012 | 7.40E-06 | -0.099 | 0.294 | 0.737 |
| 6 | rs12192239 | DLL1 | Vitamin B6, Iron, Folate intake | T | 0.630 | -0.016 | 0.004 | 7.90E-06 | 0.195 | 0.097 | 0.045 |
| 2 | rs35280662 | AC007040.2 | Vitamin B6, Ash, Sodium intake | A | 0.035 | -0.042 | 0.009 | 8.10E-06 | 0.217 | 0.229 | 0.344 |
| 3 | rs9289799 | PFN2 | Vitamin B6, Iron, Niacin intake | G | 0.200 | 0.018 | 0.004 | 9.50E-06 | -0.015 | 0.099 | 0.883 |

KARE, Korea Association resource; SNP, single nucleotide polymorphism; MAF, minor allele frequency; SE, standard error;

Supplementary table 8. Associations of individual genetic instruments for dietary vitamin C intake levels with CKD development

| **CHR** | **SNP** | **Nearest Genes** | **Mapped phenotypes** | **Effect allele** | **MAF** | **Dietary vitamin C intake** | | | **CKD** | | |
| --- | --- | --- | --- | --- | --- | --- | --- | --- | --- | --- | --- |
|  |  |  |  |  |  | **Beta** | **SE** | **P-value** | **Beta** | **SE** | **P-value** |
| 1 | rs140394939 | PDE4B | Vitamin C, Carotene intake | A | 0.015 | -0.098 | 0.020 | 1.20E-06 | -0.117 | 0.380 | 0.758 |
| 2 | rs16846116 | LRP1B | Vitamin C, Weight, BMI | A | 0.070 | 0.044 | 0.009 | 1.20E-06 | 0.129 | 0.159 | 0.416 |
| 8 | rs147902155 | SNAI2 | Vitamin C, Potassium, Vitamin B2 intake | A | 0.029 | -0.068 | 0.014 | 2.00E-06 | -0.308 | 0.306 | 0.315 |
| 1 | rs12031723 | PTBP2 | Vitamin C, Fiber, Potassium intake | C | 0.250 | -0.026 | 0.006 | 2.90E-06 | -0.129 | 0.101 | 0.201 |
| 5 | rs76463900 | HDAC3 | Vitamin C intake, WBC, Height | A | 0.140 | 0.032 | 0.007 | 4.60E-06 | -0.041 | 0.120 | 0.734 |
| 5 | rs10074128 | PRLR | Vitamin C, A, Folate intake, BUN | G | 0.390 | 0.022 | 0.005 | 6.90E-06 | 0.067 | 0.083 | 0.415 |
| 7 | rs2521745 | NPVF | Vitamin C, Fiber, vitamin B6, carotene intake | A | 0.800 | 0.026 | 0.006 | 7.30E-06 | 0.036 | 0.131 | 0.785 |
| 2 | rs3828277 | TNS1 | Vitamin C, Potassium intake, HbA1C | T | 0.065 | -0.042 | 0.009 | 7.50E-06 | -0.101 | 0.153 | 0.508 |
| 8 | rs146782291 | C8orf37 | Vitamin C, Fiber intake, Colorectal cancer | A | 0.044 | 0.052 | 0.012 | 8.90E-06 | 0.203 | 0.256 | 0.427 |
| 1 | rs898833 | STUM | Vitamin C, B6, fiber intake | T | 0.230 | -0.024 | 0.006 | 9.40E-06 | -0.083 | 0.100 | 0.406 |

KARE, Korea Association resource; SNP, single nucleotide polymorphism; MAF, minor allele frequency; SE, standard error; BUN, Blood Urea Nitrogen; BMI, Body mass index; WBC, White Blood Cell; HbA1C, Glycated Hemoglobin;

Supplementary table 9. Mendelian randomization results for the effects of dietary phosphorus, vitamin B2, B6 and C intake levels on CKD development

| **Micronutrient levels** | **SNPs** | **BETA** | **SE** | **p-value** |
| --- | --- | --- | --- | --- |
| **Dietary phosphorus intake levels** |  |  |  |  |
| IVW | 19 | 0.602 | 1.340 | 6.53E-01 |
| IVW radical | 19 | 0.602 | 1.248 | 6.29E-01 |
| MR Egger | 19 | 0.117 | 3.732 | 9.75E-01 |
| Penalised weighted median | 19 | 1.254 | 1.921 | 5.14E-01 |
| Weighted median | 19 | 1.254 | 1.836 | 4.95E-01 |
| Simple median | 19 | 1.293 | 1.933 | 5.04E-01 |
| **Dietary Vitamin B2 intake levels** |  |  |  |  |
| IVW | 19 | 2.725 | 1.151 | 1.78E-01 |
| IVW radical | 19 | 2.725 | 0.639 | 2.00E-01 |
| MR Egger | 19 | 2.509 | 3.136 | 4.34E-01 |
| Penalised weighted median | 19 | 1.990 | 1.474 | 1.77E-01 |
| Weighted median | 19 | 1.990 | 1.522 | 1.91E-01 |
| Simple median | 19 | 2.074 | 1.501 | 1.67E-01 |
| **Dietary Vitamin B6 intake levels** |  |  |  |  |
| IVW | 12 | -3.769 | 1.554 | 1.53E-02 |
| IVW radical | 12 | -3.771 | 0.965 | 9.34E-05 |
| MR Egger | 12 | -3.417 | 3.085 | 2.93E-01 |
| Penalised weighted median | 12 | -3.979 | 1.932 | 3.94E-02 |
| Weighted median | 12 | -3.979 | 1.972 | 4.36E-02 |
| Simple median | 12 | -3.977 | 1.940 | 4.04E-02 |
| **Dietary Vitamin C intake levels** |  |  |  |  |
| IVW | 10 | 2.572 | 1.275 | 4.37E-02 |
| IVW radical | 10 | 2.573 | 0.592 | 1.41E-05 |
| MR Egger | 10 | 2.092 | 3.439 | 5.59E-01 |
| Penalised weighted median | 10 | 2.952 | 1.621 | 6.86E-02 |
| Weighted median | 10 | 2.952 | 1.626 | 6.94E-02 |
| Simple median | 10 | 2.997 | 1.538 | 5.14E-02 |

CKD, chronic kidney disease; SE, standard error; Inverse variance weighted, IVW; MR, Mendelian randomization

Supplementary table 10. Results of pleiotropy test for the association between dietary micronutrients levels and CKD development

| **Dietary micronutrients levels** | SNPs, n | Intercept | SE | p-value | MR-PRESSO p-value |
| --- | --- | --- | --- | --- | --- |
| Phosphorus | 19 | 0.010 | 0.072 | 0.890 | 0.872 |
| Vitamin B2 | 19 | 0.005 | 0.069 | 0.941 | 0.970 |
| Vitamin B6 | 12 | -0.009 | 0.074 | 0.897 | 0.986 |
| Vitamin C | 10 | 0.016 | 0.111 | 0.884 | 0.895 |

SNP, single nucleotide polymorphism;


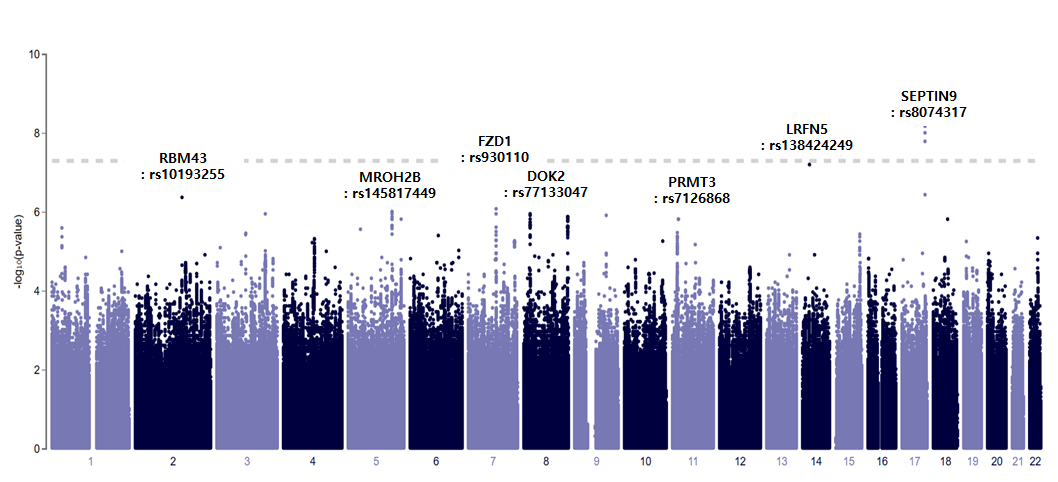


Supplementary Figure 1. A Manhattan plot of SNPs for dietary phosphorus intake levels using GWAS analysis from KARE study.


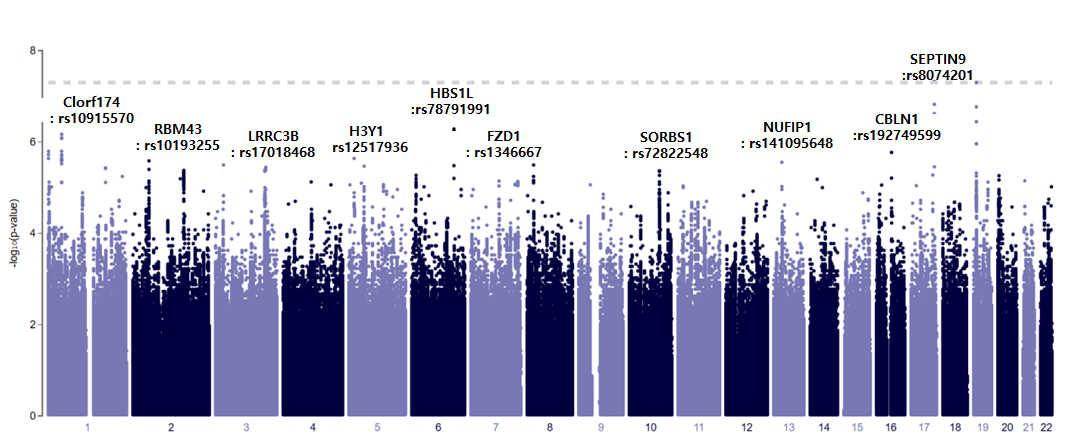


Supplementary Figure 2. A Manhattan plot of SNPs for dietary vitamin B2 intake levels using GWAS analysis from KARE study.


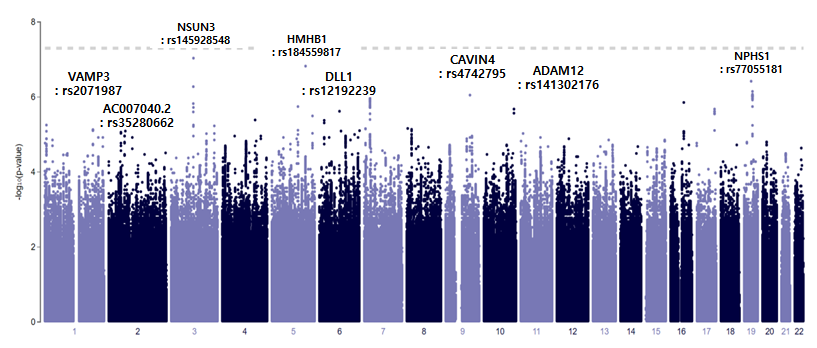


Supplementary Figure 3. A Manhattan plot of SNPs for dietary vitamin B6 intake levels using GWAS analysis from KARE study.


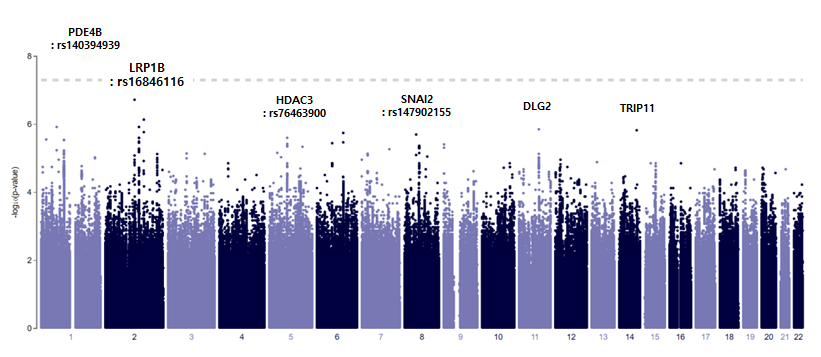


Supplementary Figure 4. A Manhattan plot of SNPs for dietary vitamin C intake levels using GWAS analysis from KARE study.


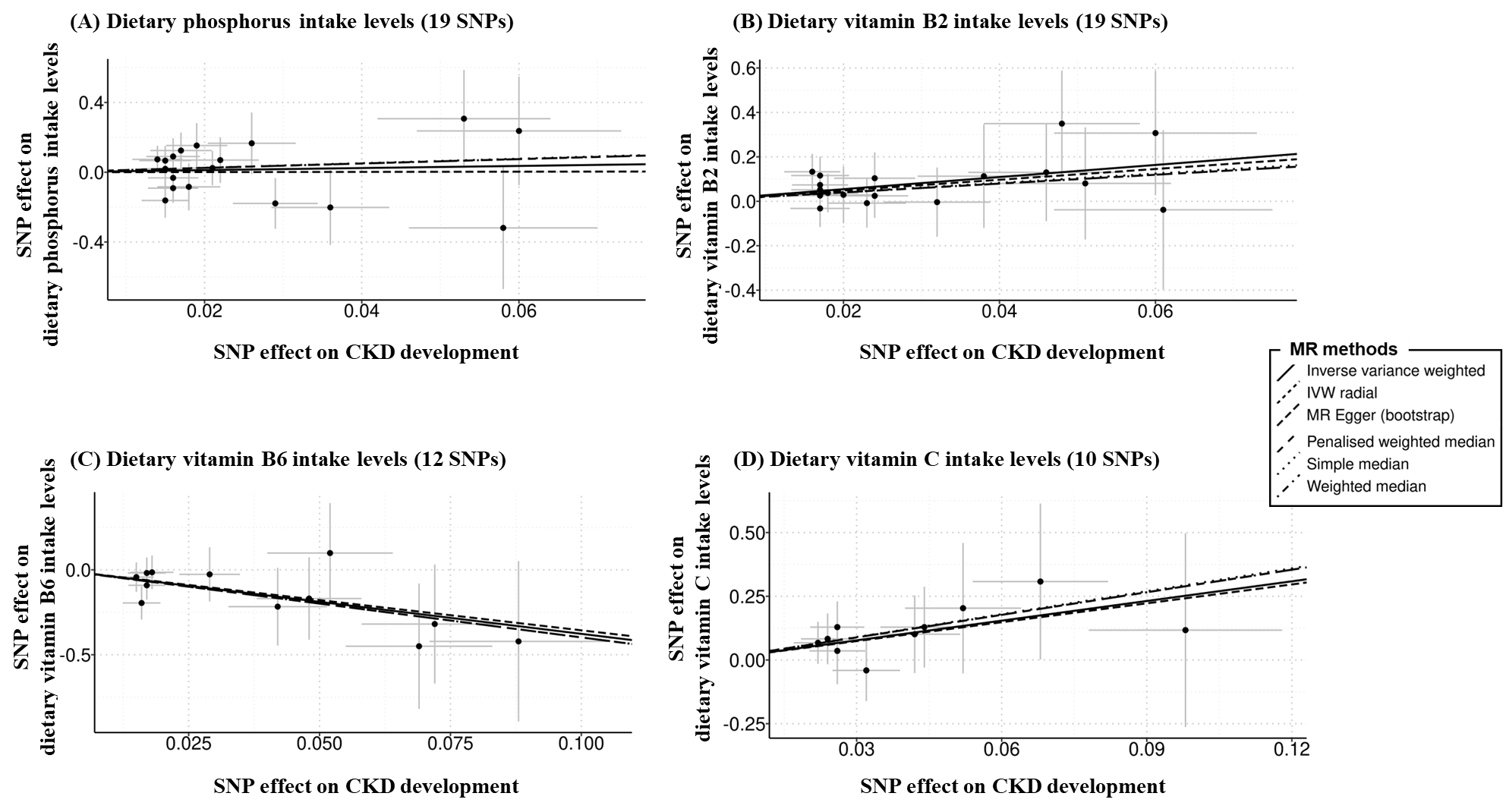


Supplementary Figure 5. Scatter plots comparing MR analyses of the associations between dietary micronutrient levels and CKD development. Associations between genetic IVs for dietary micronutrient levels and CKD development were analyzed using different MR methods. MR, Mendelian randomization; CKD, chronic kidney disease; IV, instrumental variable; SNP, single nucleotide polymorphism.


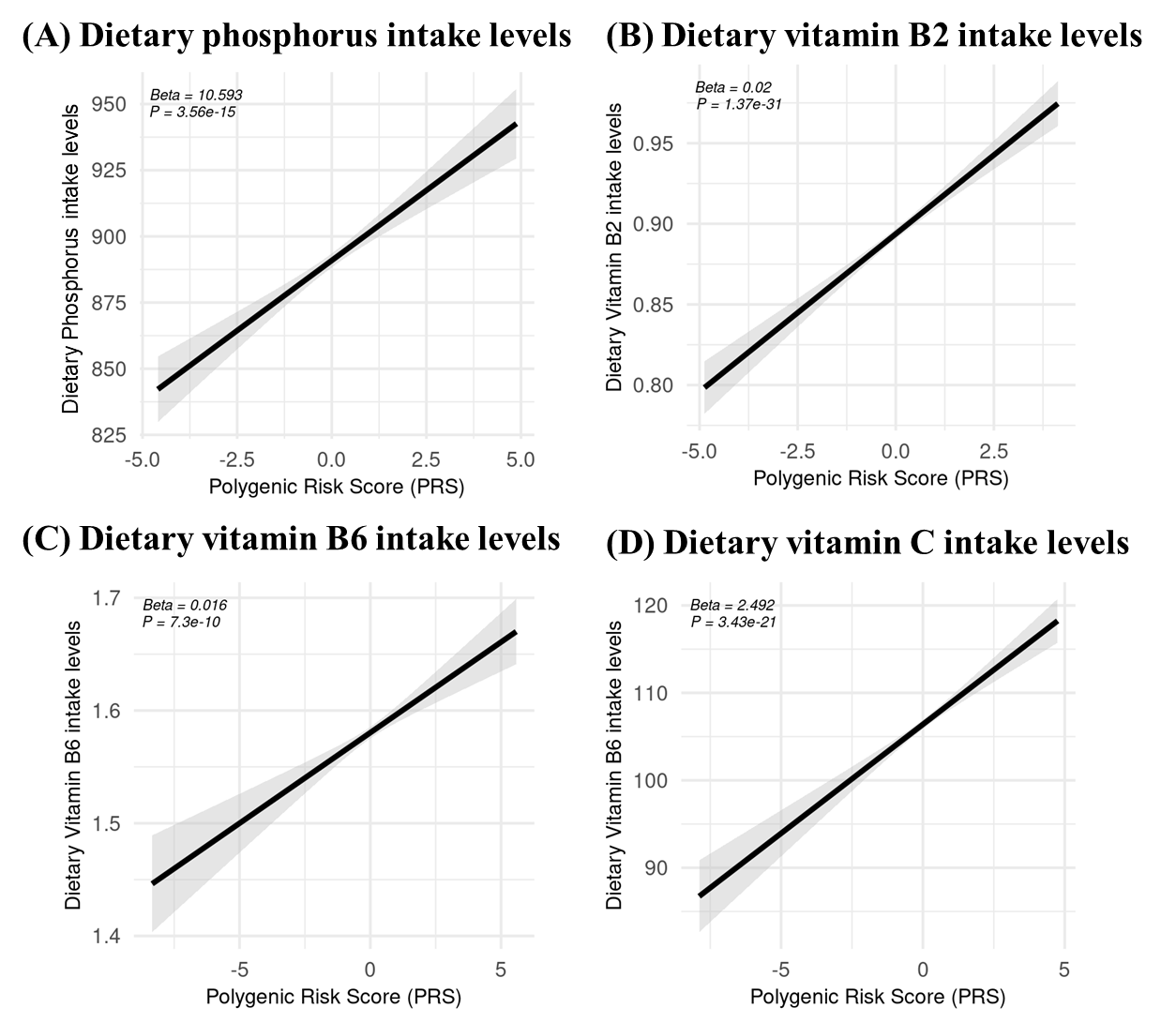


Supplementary Figure 6. Associations between the PRS derived from IVs and dietary micronutrient levels, based on linear regression models. Black lines indicate beta coefficients, and grey shaded areas represent their 95% CIs. IV, instrumental variable; PRS, polygenic risk score; CI, confidence interval.

| **(A) Dietary phosphorus intake levels** | **(B) Dietary vitamin B2 intake levels** |
| --- | --- |
| 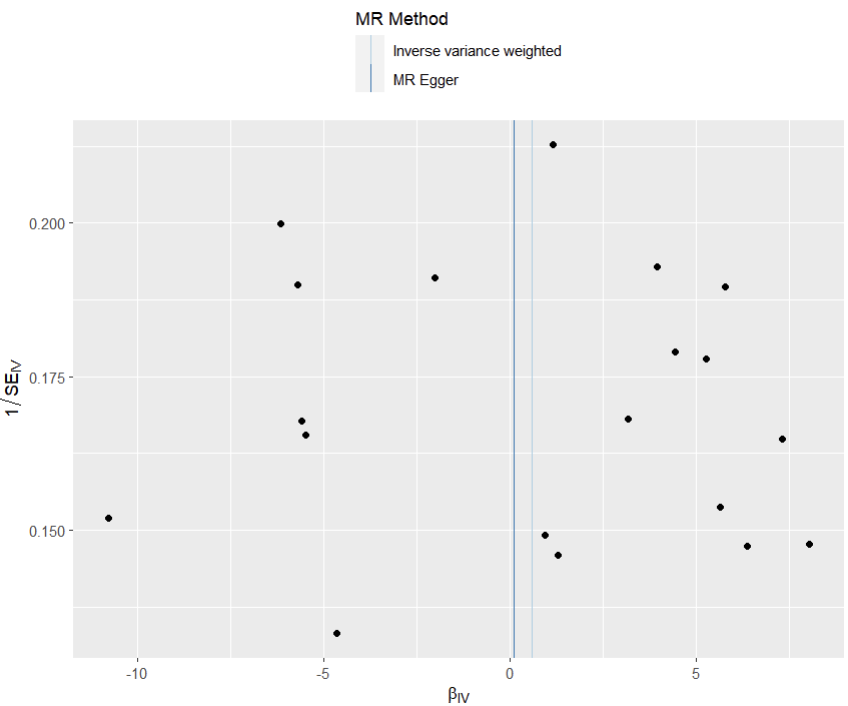 | **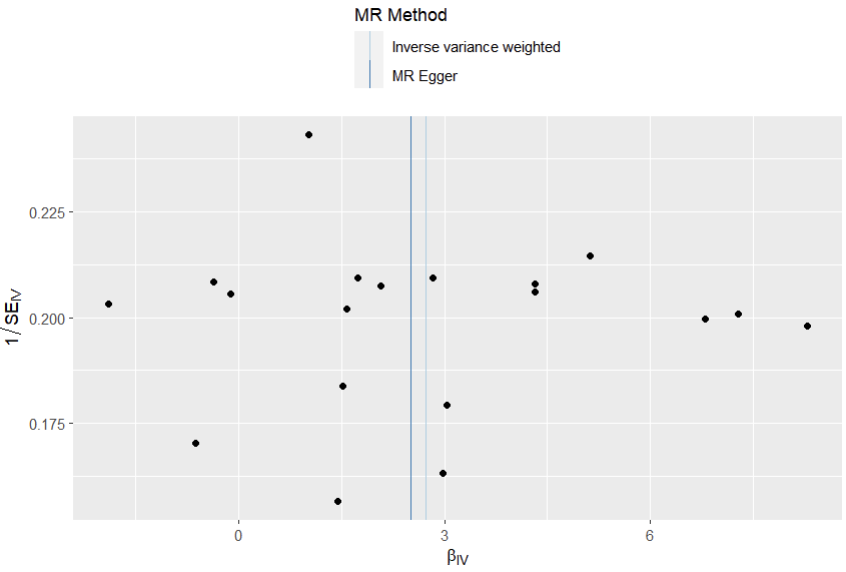** |
| **(C) Dietary vitamin B6 intake levels** | **(D) Dietary vitamin C intake levels** |
| 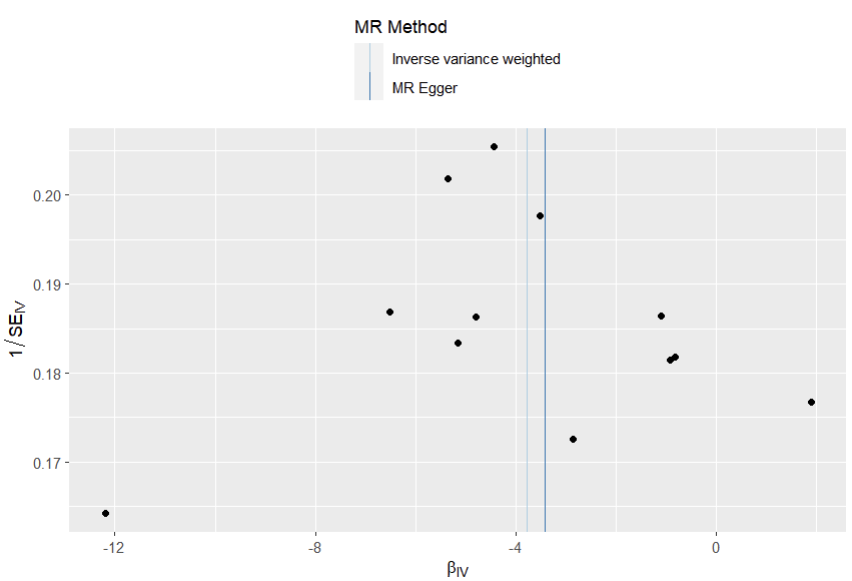 | 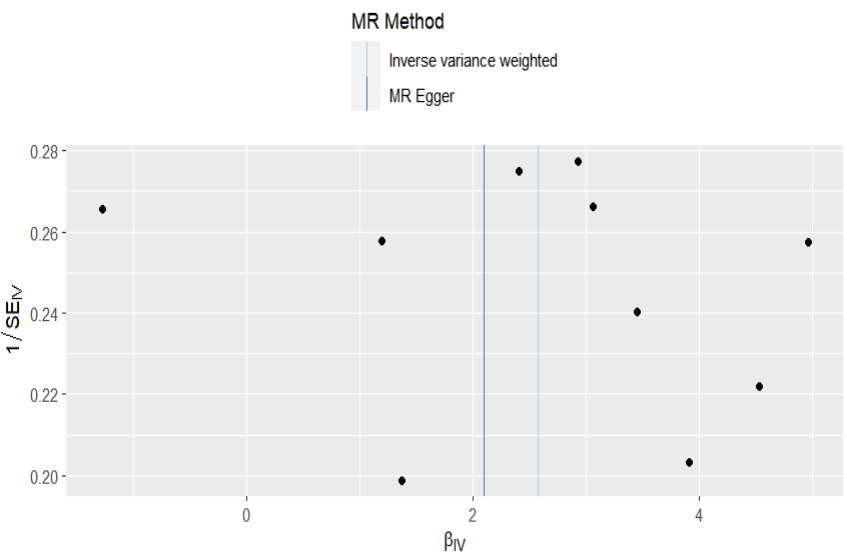 |

Supplementary Figure 7. Funnel Plot of dietary micronutrient intake levels and CKD development.
